# Wetland degradation promotes soil P fraction transformation by altering P-cycling functional genes and metabolic pathways

**DOI:** 10.3389/fmicb.2025.1677320

**Published:** 2025-11-12

**Authors:** Yumeng Jiang, Yu Zou, Miaojia Sun, Weihong Zhu, Wanling Xu

**Affiliations:** College of Geography and Ocean Sciences, Yanbian University, Hunchun, China

**Keywords:** wetland degradation, P fractions, P cycling, metabolic pathways, functional genes

## Abstract

Global wetlands have undergone varying degrees of degradation due to intense disturbances from global climatic and environmental changes, and human activities such as overgrazing and drainage. While wetland degradation is known to alter soil physicochemical properties and phosphorus (P) cycling, the mechanism governing its effects on soil P fraction transformation and P metabolism remains poorly understood. To address this, we investigated how different stages of wetland degradation—non-degraded (ND), slightly degraded (LD), moderately degraded (MD), and heavily degraded (HD)—affect soil P fractions in temperate wetlands. We analyzed soil properties, P-cycling microbial communities, functional genes, and metabolic products, employing the modified Hedley P fractionation method to elucidate clear trends in P fraction contents. Our results show that total inorganic P content decreased significantly with increasing degradation intensity. Specifically, labile Pi (Resin-Pi and NaHCO₃-Pi), mod-labile Pi (NaOH-Pi), and stable Pi (1 M HCl-Pi and Residual-P) all declined significantly, although Conc. HCl-Pi exhibited an initial decrease followed by an increase. In contrast, total organic P content increased, with significant increases in labile Po (NaHCO_3_-Po) and mod-labile Po (NaOH-Po), while stable Po (Conc. HCl-Po) decreased markedly. These shifts indicate that wetland degradation promotes the interconversion among labile P, mod-labile P, and stable P forms. The degradation process is initiated by a reduction in soil moisture, which subsequently regulates soil pH and other physicochemical properties. These changes further drive shifts in microbial community diversity, influence the abundance of P-cycling functional genes, and alter P metabolic pathways, ultimately affecting both the speciation and total pool of soil phosphorus. The accumulation of labile Po is primarily attributed to the obstruction of mineralization, resulting from the reduction of terminal functional genes in the Po mineralization pathway. These findings enhance our understanding of P-cycling mechanisms in degraded wetlands and provide a theoretical basis for phosphorus management during wetland restoration efforts.

## 1 Introduction

As the third most abundant essential nutrient in soil, following carbon and nitrogen, phosphorus (P) is critical for supporting the growth and development of both plants and microorganisms. P cycling is essential to the nutrient dynamics of wetland ecosystems (Cheesman et al., 2014; Hu et al., 2022b; Kour et al., 2021). In natural ecosystems, P cycling differs fundamentally from carbon and nitrogen cycling because it lacks a significant atmospheric gas phase. Instead, P is primarily sequestered in rocks and soils, and its mobilization depends on geological weathering and biological processes (Hong et al., 2025). The P that can be directly absorbed and utilized by plants represents the amount of P available in an ecosystem. This P determines the P utilization of that ecosystem. Therefore, P is often considered the “limiting element” in soil nutrients, and its availability directly affects primary productivity and ecosystem functioning in wetlands (Lie et al., 2022). In the soil P cycle, P is converted to soluble forms and taken up by plants or microorganisms, and then returned to the soil after the organisms die (Kruse et al., 2015). To better study the composition and bioavailability of soil P, it is categorized into a series of organic and inorganic fractions. These P fractions differ in their turnover, transformation, and mobility in the soil, reflecting variations in their biological availability. Based on solubility, Hedley et al. classified P into several forms: labile P (Resin-P, NaHCO₃-Pi, NaHCO₃-Po), moderately labile P (NaOH-Pi, NaOH-Po), and stable P (1 M HCl-P, conc. HCl-Pi, conc. HCl-Po, Residual-P) (Hedley et al., 1982; Weihrauch and Opp, 2018). This classification is currently the most widely used comprehensive P fractionation method. Changes in soil P fractions are influenced by many factors, including soil physicochemical properties, nutrient status, enzyme activity, and microbial mineralization-immobilization processes (Inamdar et al., 2017; Six et al., 2004). Among these, soil pH is a fundamental factor affecting P speciation and bioavailability (Dick et al., 1983). Some studies have shown that high concentrations of Al and Fe ions in acidic soils enhance the complexation capacity of P with these metal ions (Coolen et al., 2011). Research has found that drought-induced increases in pH can significantly reduce the content of calcium phosphates in temperate forests, accompanied by an increase in inorganic and organic P bound to secondary minerals (Fe/Al oxides) (Zhang et al., 2020). Simultaneously, the oxidative conditions following wetland drainage can increase the abundance of P-solubilizing microorganisms in the soil, leading to elevated levels of labile P and AP (Jiang et al., 2024). Additionally, changes in soil organic matter composition can alter microbial biomass, activity, and community structure, thereby affecting phosphorus (P) forms and availability (Bai et al., 2023; Wu et al., 2025). In summary, existing studies have demonstrated that soil environmental changes can significantly affect P fractions. It has been confirmed that wetland degradation, as a typical environmental disturbance, can strongly alter soil physicochemical properties and thereby profoundly influence the P cycling process (Bergkemper et al., 2016). However, the specific mechanisms through which wetland degradation drives the transformation of P fractions still lack systematic and in-depth investigation.

Wetland degradation significantly alters soil environments, initiating shifts in microbial community structure (Hu et al., 2022b; Liang et al., 2020). These microbial communities are essential for soil P cycling. Specifically, soil microorganisms, especially P-solubilizing microorganisms, are key actors in four core P cycling processes: solubilization of inorganic phosphorus (Pi), mineralization of organic phosphorus (Po), regulation of P starvation response, and P uptake/transport (Bergkemper et al., 2016; Dai et al., 2020). They primarily function by secreting organic acids and enzymes to hydrolyze, dissolve, or mineralize insoluble P compounds (Pang et al., 2024; Rawat et al., 2020). Wetland degradation commonly causes a decline in groundwater level, reduced soil water content, and higher redox potential (Cui et al., 2020). As soil moves from reducing to oxidizing conditions, microbial communities engaged in Pi solubilization and Po mineralization may become more active, thereby accelerating P transformation (Su et al., 2017; Wang et al., 2017). Furthermore, recent studies show that rising soil pH in degraded wetlands can strongly impact functional microorganisms in the P cycle, further enhancing P cycling efficiency and increasing soil AP (Li et al., 2022). Conversely, wetland degradation often reduces soil organic carbon (Zheng et al., 2024). As a vital energy source, decreased organic carbon content may hinder the growth of microbes preferring rich organic carbon environments (Lehmann et al., 2017; Wang et al., 2016). This decline can reduce microbial diversity, especially key groups like *phoD* gene-containing bacteria, ultimately disrupting normal soil P cycling (Cui et al., 2025). In summary, wetland degradation may directly affect microbial community structure and function by altering soil physicochemical properties and organic carbon content, profoundly influencing soil P cycling.

Wetlands are ecosystems found between land and water bodies. They have unique soil, hydrological, and biological characteristics and provide many ecological services locally and globally, which makes them crucial for biodiversity conservation, nutrient cycling, climate regulation, water conservation, and human health. They are often referred to as the “kidneys of the earth” and the “biological gene pool” (Wu et al., 2021). Global climate change and human activities, such as overgrazing and drainage, have strongly affected wetlands. As a result, wetland areas have significantly decreased worldwide, with approximately 50% degraded or lost. This loss has led to severe ecological and social problems (Jiang et al., 2017; Meng et al., 2017). As one of the limiting nutrients for primary productivity in wetlands, P promotes plant growth and ensures normal ecological functions of wetland systems (Xu et al., 2022). Current wetland P research focuses on two main approaches: first, analyzing P storage in aquatic plants and adsorption–desorption characteristics of sediment P within water-land transition zones to investigate P transformation in coastal wetland (Bai et al., 2017; Berthold et al., 2018; Karstens et al., 2015); second, analyzing changes in wetland soil P-cycling microorganisms and functional genes to assess P availability and transformation sources (Hu et al., 2022a; Liu et al., 2023; Wu et al., 2025). Only a few studies have examined the combined influence of soil P fraction dynamics and microbial functional genes involved in P cycling on wetland soil P availability, and these have mostly been in alpine wetlands (Zhu et al., 2017). In contrast, there is a knowledge gap regarding microbial mechanisms and metabolic pathways that drive P fraction transformation during temperate wetland degradation. Wetland degradation is a typical process driven by the combined effects of natural and anthropogenic factors. Investigating the driving mechanisms and response patterns of wetland ecosystems can help us better understand their vulnerability, adaptability, and tipping points.

The Jingxin Wetland, a significant transboundary temperate wetland ecosystem situated at the northeastern tip of China, exhibits composite characteristics of both inland and estuarine wetlands.” It serves as a critical stopover and breeding site for migratory birds within the Northeast Asia flyway (Liu et al., 2021a). In recent years, anthropogenic activities such as overgrazing and agricultural pollution have led to substantial biodiversity loss and severely compromised ecosystem stability in this region. Wetland degradation has further disrupted nutrient cycling processes, exacerbating ecological imbalance (Liu et al., 2021a; Zheng et al., 2017). The degradation reflects common pressures faced by temperate composite wetlands worldwide, including hydrological alteration due to human disturbance, habitat fragmentation, and non-point source pollution. The degradation mechanisms observed here provide a theoretical and empirical basis for understanding similar wetland ecosystems. Moreover, located at the junction of China, Russia, and the Democratic People’s Republic of Korea (DPRK), the Jingxin Wetland represents a model system for studying transboundary ecological degradation and cooperative conservation. Its strategic position within a migratory bird corridor underscores the broad spatial implications and potential cascading effects of its degradation. Investigating changes in P fractions during wetland degradation in this area will help elucidate how temperate wetland decline affects key pathways of P cycling and provide a scientific foundation for transboundary wetland restoration. Such insights are crucial for advancing regional ecological integrity and facilitating cross-border environmental governance. Previous studies have reported a significant decrease in total soil P content with intensifying degradation in this wetland (Zheng et al., 2017). However, changes in individual P fractions and the underlying microbial mechanisms driving P transformation remain poorly understood. Therefore, our specific objectives were to: (1) determine the impact of wetland degradation on the quantity of soil phosphorus components; (2) assess the effects of phosphorus-related functional genes and phosphorus metabolic pathways during wetland degradation processes; (3) clarify key regulators and mechanisms underlying the effects of governing soil phosphorus content in wetland degradation processes. We propose the following hypotheses: (1) Wetland degradation significantly reduces total P content and promotes a shift in P speciation from stable, insoluble forms (e.g., HCl-P) toward labile, soluble forms (e.g., Resin-P and AP); (2) Degradation restructures the soil microbial community, leading to altered abundance and composition of P-cycling functional genes, particularly increase in those involved in Po mineralization and Pi solubilization; (3) Soil water content, pH, and organic matter are the primary environmental factors governing P transformations, indirectly modulating the abundance of key microbial hosts (e.g., *Actinobacteria* and *Proteobacteria*) of P-cycling functional genes by shaping microbial community and soil redox conditions.

## 2 Materials and methods

### 2.1 Study sites

This study was conducted in the Jingxin Wetland (42°27′-42°40′N, 130°25′-130°39′E) in the lower reaches of the Tumen River, located in Yanbian Korean Autonomous Prefecture, Jilin Province, Northeast China. It is a tri-border area shared by China, Russia, and the DPRK. Influenced by the Sea of Japan, the Jingxin Wetland experiences monsoons in spring and fall, has a mild and humid climate, frequently cloudy skies with low sunshine, an average annual temperature of 5.6 °C, and an average annual rainfall of 823.7 mm. It falls within the mid-temperate zone near the coast with a monsoon climate zone. Previous studies identify the Jingxin Wetland as a complex comprising riverine wetlands, lake wetlands, marsh wetlands, and artificial wetlands, with numerous rivers and lakes. The area includes approximately 8,000 ha of watery swamps and is rich in plant and animal resources.

### 2.2 Experimental design

According to the characteristics of the plant community structure and soil physicochemical properties of the wetland and the basic investigation of the sample plots, combined with the laboratory’s previous research experience and results, and according to the different degrees of degradation the herbaceous swamp wetland, four treatments were randomly set up: non-degraded (ND), slightly degraded (LD), moderately degraded (MD), and heavily degraded (HD). The sample plot area of the sample plot is 5 m × 5 m, and each treatment was set up with six replicates, totaling 24 sample plots. The basic profiles of different degraded wetlands are shown in Supplementary Table S1. Then, soil was randomly sampled from a depth of 0–10 cm at five points using the five-point method with a soil auger. After removing debris (e.g., stones and roots), the five subsamples were homogenized to form one composite sample and sieved through a 2-mm mesh. A total of 24 composite samples were prepared. Each soil sample was divided into three parts: one was put into a freezing tube and stored in liquid nitrogen for metagenome and non-targeted metabolome testing; one was put into a refrigerator at −20 °C for total nitrogen, total carbon, total P and other indicators of the soil; and one was naturally air-dried for soil pH and P components and other indicators of the test. At the same time, soil samples were collected using the ring knife method to measure soil bulk density and water content.

### 2.3 Soil P fractions

A modified Hedley P fractionation was conducted for the P fraction, following the procedure described by Waldrip et al. (2011). Based on their method, 0.5 g of soil was weighed into a 50 mL tube and then deionized H_2_O (30 mL), 0.5 M NaHCO_3_ (30 mL), 0.1 M NaOH (30 mL), 1 M HCl (diluted HCl, 30 mL), and concentrated HCl were successively added for sequential extractions. The soil P pools were classified into nine P fractions, including labile-P fractions (Resin-P, NaHCO_3_-Pi, and NaHCO_3_-Po), moderately labile-P fractions (NaOH-Pi and NaOH-Po), and stable-P fractions (Diluted HCl-P [1 M HCl-Pi], Concentrated HCl-Pi [Conc. HCl-Pi], Concentrated-HCl-Po [Conc. HCl-Po], and Residue-Pi). Soil Pi content was calculated by summing the contents of Resin-P, NaHCO_3_-Pi, NaOH-Pi, 1 M HCl-Pi, Conc. HCl-Pi, and Residual-P. Soil Po content was calculated by summing the contents of NaHCO_3_-Po, NaOH-Po, and Conc. HCl-Po.

### 2.4 Soil physicochemical measurements

Soil pH was measured in a 1:2.5 soil–water suspension with a pH meter (Mettler Toledo, Shanghai, China). Soil water content (SW) was determined using the oven-drying method, calculated as SW = (fresh soil weight – dry soil weight) / fresh soil weight × 100%. Soil total carbon (TC) and total nitrogen (TN) contents were measured using an elemental analyzer (Vario EL cube, Elementar, Germany). Soil inorganic nitrogen fractions (NH_4_^+^-N and NO_3_^−^-N) were quantified by continuous flow analysis. Available phosphorus (AP) was extracted using 7.5 mol L^−1^ NaHCO_3_ solution (pH 8.5) and subsequently determined. Dissolved organic carbon (DOC) content was analyzed using a TOC analyzer (Elementar vario TOC select, Elementar Analysensysteme GmbH, Hanau, Germany). Microbial biomass phosphorus (MBP) was measured by the chloroform fumigation-extraction method.

### 2.5 Soil DNA extraction and sequencing

0.2 g of stool /soil material was used to extract total genomic DNA with the E. Z. N. A.® soil DNA Kit (Omega Bio-tek, Norcross, GA, United States) according to the manufacturer’s instructions. The concentration and purity of extracted DNA were determined using SynergyHTX and NanoDrop2000, respectively. DNA quality was checked on 1% agarose gel. The DNA extract was fragmented to an average size of approximately 350 bp using a Covaris M220 (Gene Company Limited, China) for paired-end library construction. Paired-end library was constructed using NEXTFLEX Rapid DNA-Seq (Bioo Scientific, Austin, TX, United States). A paired-end sequencing was performed on Illumina NovaSeq™ X Plus (Illumina Inc., San Diego, CA, United States) at Majorbio Bio-Pharm Technology Co., Ltd. (Shanghai, China) using the NovaSeq X Series 25B Reagent Kit according to the manufacturer’s instructions.^1^ The metagenomic sequencing data associated with this project have been deposited in the NCBI Short Read Archive database.

The data were analyzed on the free online platform of the Majorbio Cloud Platform.^2^ The raw sequences were used to get clean reads. First, the reads that contained adapters were entirely removed. Second, the reads containing N (uncertain base) greater than 1% were removed. Third, low-quality reads (Q ≤ 20) with contents greater than 50% were removed.

The quality-filtered data were assembled using MEGAHIT.^3^ Contig with a length ≥ 300 bp were selected as the final assembling result. Open reading frames (ORFs) from each assembled contigs were predicted using Prodigal (Li et al., 2015),^4^ and ORFs with a length of ≥ 100 bp were retrieved. A non-redundant gene catalog was constructed using CD-HIT (Fu et al., 2012)^5^ with 90% sequence identity and 90% coverage. Gene abundance for a certain sample was estimated by SOAPaligner (Li et al., 2008)^6^ with 95% identity.

The amino acid sequences of the non-redundant gene set were aligned to the NR and KEGG databases using Diamond (Buchfink et al., 2014)^7^ (BLASTP alignment parameters set expectation e-value to 1e-5). Species annotations and KEGG functions corresponding to the genes were obtained. The abundance of the corresponding functional categories was calculated using the sum of the abundance of the genes corresponding to KO, Pathway, EC, and Module.

In total, 103 soil P cycle genes with their corresponding KO numbers were searched in the datasets based on previous publications (Li et al., 2022; Liu et al., 2023). They were classified into four categories according to their functions in the soil P cycles based on previous studies (Dai et al., 2020; Hartman et al., 2017; Ma Q. et al., 2020). The KO numbers, gene names, functions, and classifications of the genes associated with soil P cycling are shown in Supplementary Table S3.

### 2.6 Statistical analyses

Statistical analyses were conducted using SPSS 26.0. One-way analysis of variance (ANOVA) was performed to evaluate significant differences in P fractions, functional gene abundance, and environmental factors across wetland degradation levels. When the assumption of homogeneity of variance was met (Levene’s test, *p* > 0.05), Tukey’s honestly significant difference (HSD) *post hoc* test was applied for multiple comparisons. In cases where the homogeneity of variance assumption was violated (*p* ≤ 0.05), Tamhane’s T2 test was used. For data that deviated from a normal distribution (Shapiro–Wilk test, *p* ≤ 0.05), the Kruskal-Wallis non-parametric test was employed.

Spearman’s rank correlation analysis was carried out in R software (version 4.1.0) to assess associations among P-cycling functional genes, with significant correlations defined as |*r*| > 0.7 and *p* < 0.05 being selected for further analysis. Redundancy analysis (RDA) was implemented with the “vegan” package to identify environmental factors influencing P fractions. The “randomForest” package was used to evaluate the relative importance of phosphorus cycling functional genes on P fractions, and partial least squares path modeling (PLS-PM) was developed using the “plspm” package to analyze pathways among key driving factors. All figures were generated using Origin 2021 and Microsoft PowerPoint 2019.

## 3 Results

### 3.1 Changes in P fractions and their availabilities in response to wetland degradation

**Figure 1:**
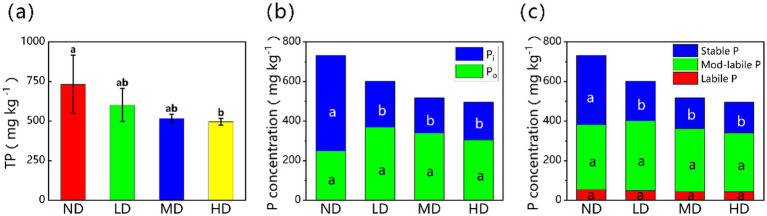
**(a)** Total phosphorus and the concentration of **(b)** Pi, Po, and **(c)** stable P, mod-labile P, and labile P fractions for different degraded wetlands. Different lowercase letters in the same row mean significant difference at *p* < 0.05 among the four treatments. Pi, inorganic phosphorus; Po, organic phosphorus; ND, non-degraded wetland; LD, slightly degraded wetland; MD, moderately degraded wetland; HD, heavily degraded wetland.

Wetland degradation processes significantly changed soil P fractions and their distribution (Figure 1). As degradation intensity increased, total phosphorus (TP) decreased by 32.4%. TP fell from 732.58 ± 183.43 mg kg^−1^ in non-degraded wetlands to 495.57 ± 21.67 mg kg^−1^ in heavily degraded wetlands. Inorganic phosphorus (Pi) also dropped sharply across the four wetland types (ND, LD, MD, HD). Their Pi concentrations were 483.13 ± 86.30, 232.64 ± 36.05, 178.11 ± 11.48, and 191.01 ± 17.04 mg kg^−1^, respectively, showing a 60.5% reduction (*p* < 0.05) with increasing degradation. In contrast, organic phosphorus (Po) first increased, then decreased. The concentrations of Po were 249.45 ± 101.91, 369.76 ± 74.57, 339.27 ± 24.22, and 304.56 ± 11.60 mg kg^−1^ in ND, LD, MD, and HD wetlands, respectively. However, these Po changes were not statistically significant (*p* > 0.05). Looking at P lability, stable P concentrations dropped significantly (*p* < 0.05) as degradation progressed. Stable P was 350.44 ± 56.37, 200.87 ± 30.99, 155.25 ± 7.64, and 155.14 ± 14.26 mg kg^−1^ in ND, LD, MD, and HD wetlands, respectively. This is a 55.7% decrease from non-degraded to heavily degraded conditions. Yet, neither labile P nor moderately labile P demonstrated significant changes during degradation (*p* > 0.05).

**Figure 2:**
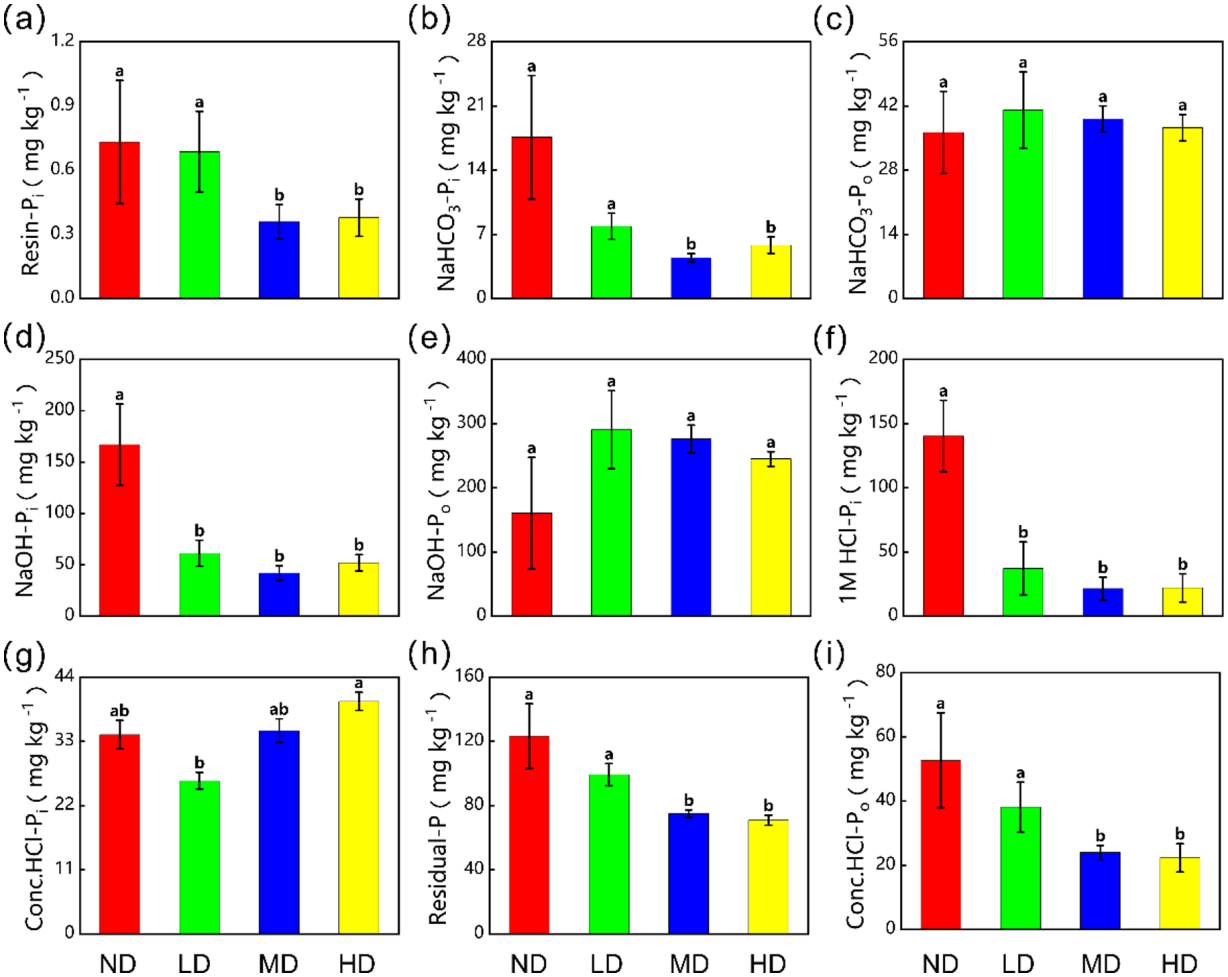
The contents of different soil P forms in the four treatments. **(a)** Resin-Pi, **(b)** NaHCO₃-Pi, **(c)** NaHCO₃-Po, **(d)** NaOH-Pi, **(e)** NaOH-Po, **(f)** 1M HCl-Pi, **(g)** Conc.HCl-Pi, **(h)** Residual-P, and **(i)** Conc.HCl-Po. Data are reported as mean ± 1 SE (*n* = 6). Different lowercase letters in the same row mean significant difference at *p* < 0.05 among the four treatments. ND, non-degraded wetland; LD, slightly degraded wetland; MD, moderately degraded wetland; HD, heavily degraded wetland.

With more severe wetland degradation, most P fractions decreased significantly (*p* < 0.05). For instance, labile Pi (Resin-Pi and NaHCO_3_-Pi) decreased from 0.73 ± 0.29 mg kg^−1^ and 17.60 ± 6.73 mg kg^−1^ in ND to 0.38 ± 0.09 mg kg^−1^ and 5.83 ± 0.9 mg kg^−1^ in HD. There are reductions of 48.3 and 66.9% (Figures 2a,b). Moderately labile Pi (NaOH-Pi) decreased from 167.08 ± 39.45 mg kg^−1^ in ND to 51.98 ± 7.99 mg kg^−1^ in HD, a 68.9% decrease (Figure 2d). Stable P fractions (1 M HCl-Pi, Conc. HCl-Po, and Residual-P) also declined. Their values were 140.34 ± 27.78 mg kg^−1^, 52.71 ± 14.77 mg kg^−1^, and 123.24 ± 20.21 mg kg^−1^ in ND. These changed to 22.10 ± 10.98 mg kg^−1^, 22.32 ± 4.41 mg kg^−1^, and 70.87 ± 3.14 mg kg^−1^ in HD. The reductions were 84.3, 57.7, and 42.5% (Figures 2f,h,i). Degradation caused changes in Conc. HCl-Pi as well. It decreased by 23.1% in slightly degraded (LD) wetlands but increased by 51.8% in HD wetlands (*p* < 0.05, Figure 2g). In contrast, Po fractions (NaHCO_3_-Po and NaOH-Po) did not change significantly across degradation stages (*p* > 0.05).

### 3.2 Changes in soil physicochemical properties in response to wetland degradation

**Figure 3:**
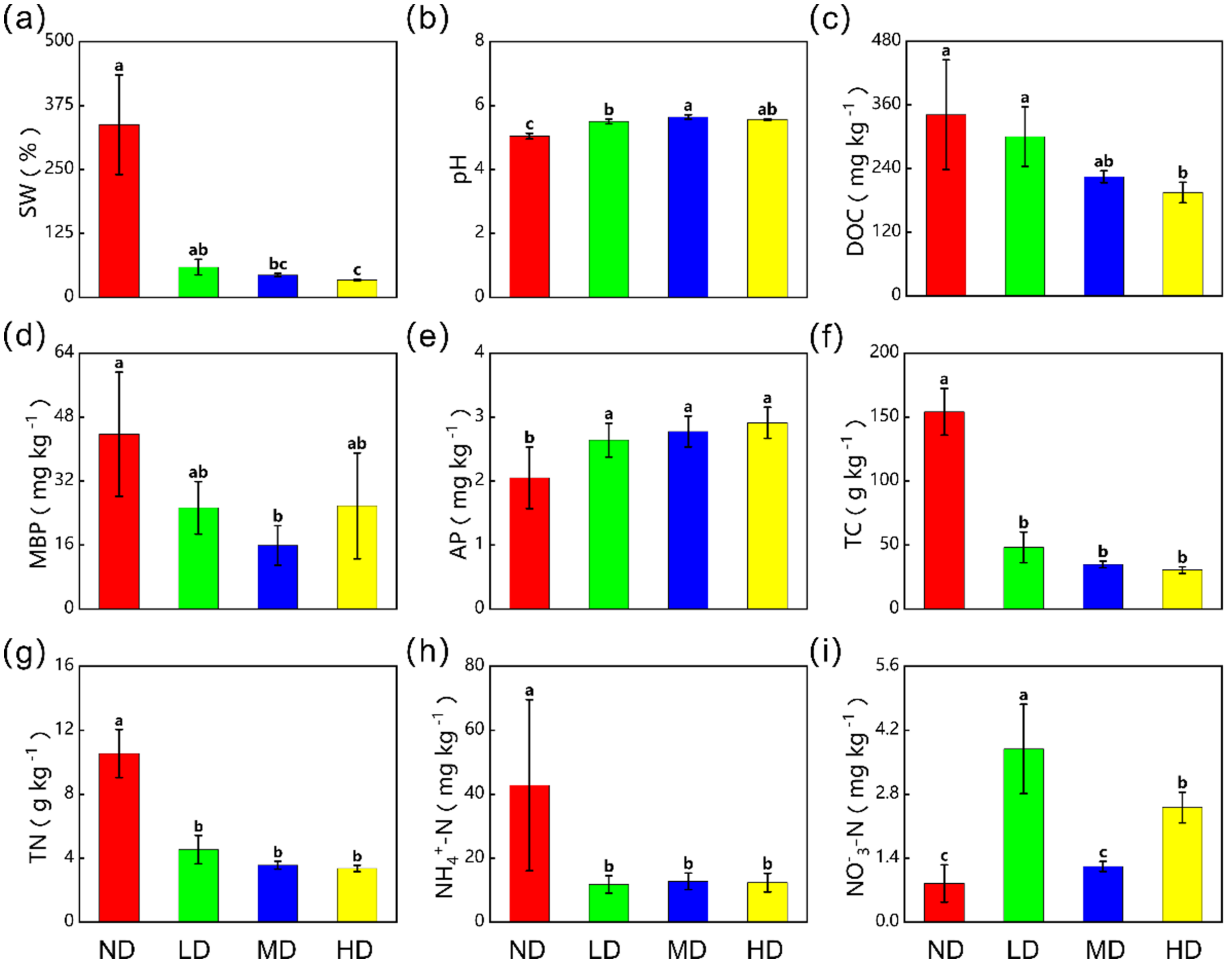
The effects of wetland degradation on **(a)** soil water content (SW), **(b)** soil pH (pH), **(c)** soil dissolved organic carbon (DOC), **(d)** microbial biomass phosphorus (MBP), **(e)** available phosphorus (AP), **(f)** total carbon (TC), **(g)** total nitrogen (TN), **(h)** ammonium nitrogen content (NH_4_^+^-N), **(i)** nitrate nitrogen content (NO_3_^−^-N). ND, non-degraded wetland; LD, slightly degraded wetland; MD, moderately degraded wetland; HD, heavily degraded wetland. Data are reported as mean ± 1 SE (*n* = 6). Different lowercase letters in the same row mean significant difference at *p* < 0.05 among the four treatments.

Wetland degradation significantly altered soil physicochemical properties (Figure 3). As degradation intensity increased from ND to HD wetlands, key soil properties changed markedly. Soil water content (SW), dissolved organic carbon (DOC), total carbon (TC), total nitrogen (TN), and ammonium nitrogen (NH₄^+^-N) decreased by 89.9, 42.9, 80.2, 68.1, and 71.1%, respectively (*p* < 0.05). Soil pH increased significantly from 5.05 ± 0.89 mg kg^−1^ in ND wetlands to 5.57 ± 0.02 mg kg^−1^ in HD wetlands. This rise represented an 11.9% increase and a shift from acidic to weakly acidic.

Notably, available phosphorus (AP) increased from 2.05 ± 0.48 mg kg^−1^ to 2.91 ± 0.24 mg kg^−1^, a rise of 41.9%. Despite this significant increase (*p* < 0.05), the absolute change in AP was small. This is because its baseline concentration was inherently low compared to the total soil P pool.

### 3.3 Changes in microbial diversity and genes involved in P cycling in response to wetland degradation

**Figure 4:**
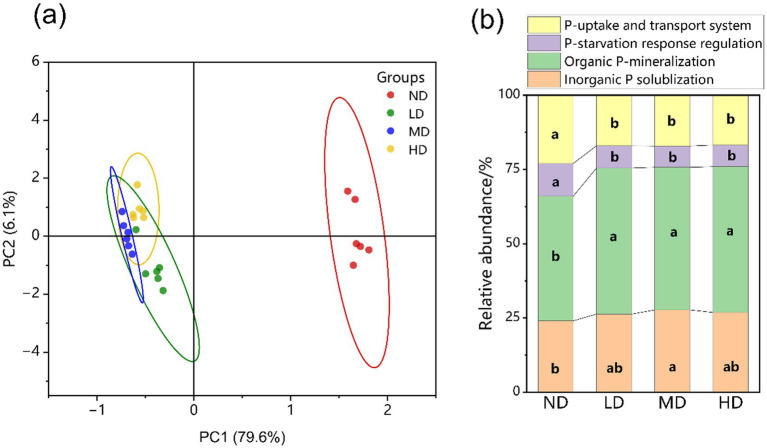
**(a)** PCA analysis of phosphorus cycling genes across wetlands with varying degradation levels. The dashed ellipses represent 95% confidence intervals. **(b)** Differential relative abundance of genes associated with the four phosphorus cycling processes. ND, non-degraded wetland; LD, slightly degraded wetland; MD, moderately degraded wetland; HD, heavily degraded wetland.

We detected a total of 103 functional genes associated with the mineralization, solubilization, transport, and regulation of P in the metagenomes. This genetic repertoire helps elucidate the microbial genetic mechanism regulating soil P cycling (Supplementary Table S4). The diversity and richness of soil microbial communities, as reflected by Shannon and Chao1 indices, were significantly higher in non-degraded wetlands than in degraded ones (Supplementary Figure S1). Furthermore, wetland degradation profoundly altered the community composition (α-diversity) of microbes harboring P-cycling genes. Principal component analysis (PCA) revealed a clear separation between non-degraded and degraded wetlands based on the profiles of P-cycling functional genes (Figure 4a).

Following wetland degradation, the relative abundance of genes involved in P uptake/transport and P starvation response regulation decreased markedly within the P-cycling functional gene pool (Figure 4b). In contrast, the proportions of genes related to Po mineralization and Pi solubilization increased significantly (*p* < 0.05).

### 3.4 Changes in the relative abundances of P-cycling functional genes in response to wetland degradation

To better understand changes in microbial P cycling across different stages of wetland degradation, the top 45 most abundant genes involved in Pi solubilization, Po mineralization, P uptake/transport system, and P-starvation response regulation were quantitatively analyzed.

**Figure 5:**
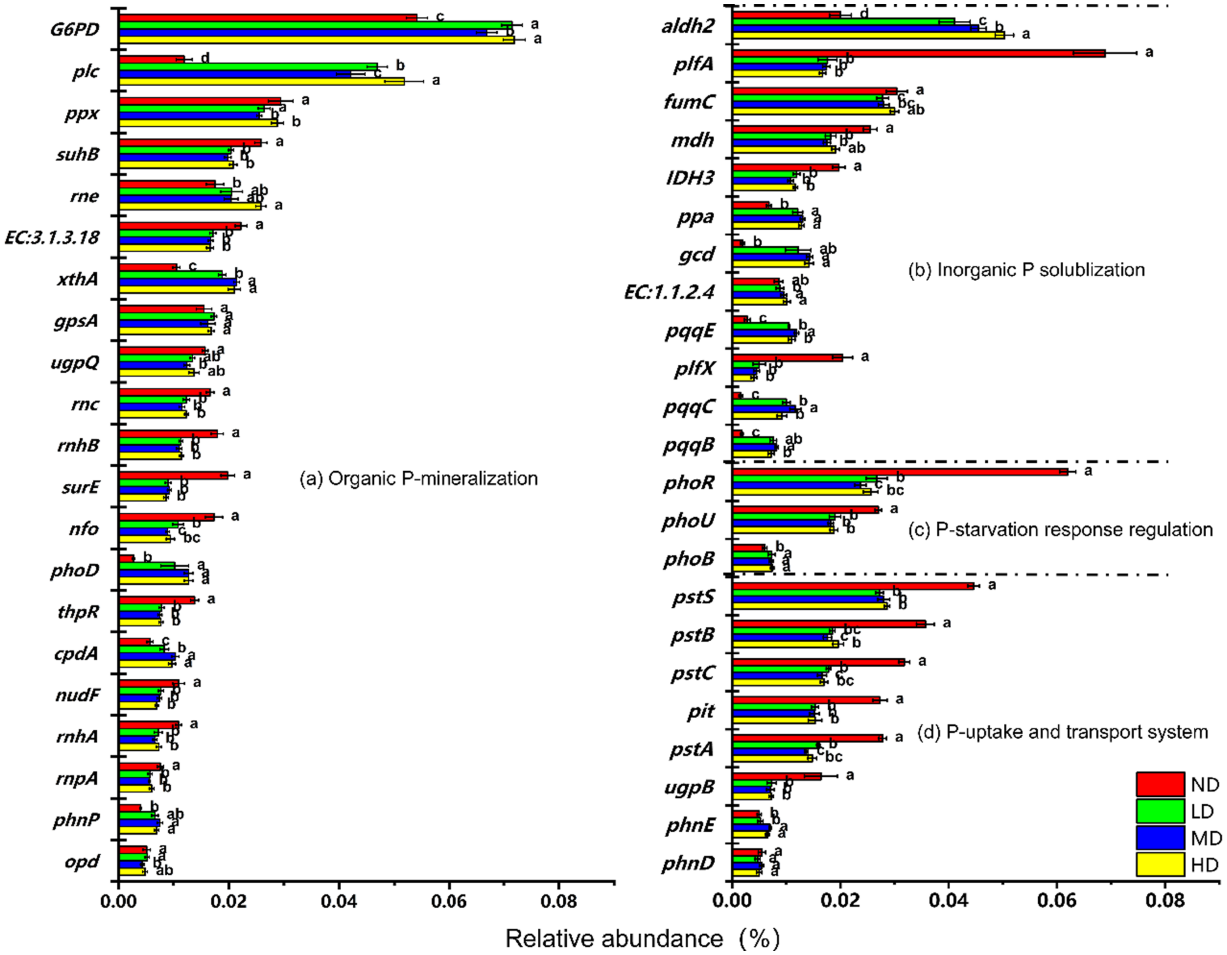
The impacts of wetland degradation on the abundances of **(a)** organic P mineralization, **(b)** inorganic P solubilization, **(c)** P starvation response regulation, and **(d)** P uptake and transport system. Data are reported as mean ± 1 SE (*n* = 6). Different lowercase letters in the same row mean significant difference at *p* < 0.05 among the four treatments. ND, non-degraded wetland; LD, slightly degraded wetland; MD, moderately degraded wetland; HD, heavily degraded wetland.

Significant variations in the relative abundance of key P-cycling genes abserved among wetland degradation stages (Figure 5). Among the functional genes associated with Po mineralization, the relative abundances of *G6PD* and *plc*—the two most abundant genes in this category—increased significantly by 33 and 335.5%, respectively, in degraded wetlands compared to non-degraded wetlands (*p* < 0.05). The relative abundance of *phoD*, another representative gene of the Po mineralization also rose significantly following degradation (*p* < 0.05). In contrast, other Po mineralization genes, including *ppx*, *rne*, *EC3.1.3.18*, and *rnc*, showed a marked decrease. Despite these divergent responses, the overall relative abundance of Po mineralization functional genes exhibited an upward trend (Figure 4).

With the Pi solubilization functional genes, *aldh2* and *plfA* displayed the highest relative abundances. Compared to non-degraded wetlands, the relative abundance of *aldh2* increased significantly by 151.7% (*p* < 0.05) in degraded wetlands, whereas that of *plfA* decreased significantly by 75.8% (*p* < 0.05). Meanwhile, other key Pi-solubilizing genes—*ppa*, *gcd*, and *pqq*—increased significantly by 87.4, 634.3, and 349.8%, respectively (*p* < 0.05). Although the *plfA* abundance declined, the substantial increases in major Pi-solubilizing genes such as *ppa*, *gcd*, and *pqq* led to a significant overall increase in the relative abundance of Pi solubilization genes. Collectively, these results demonstrate that wetland degradation significantly enhanced the relative abundance of most functional genes related to Pi solubilization (*aldh2*, *ppa*, *gcd*, *pqq*) and Po mineralization (*G6PD*, *plc*, *phoD*) (*p* < 0.05). Conversely, the relative abundance of P-starvation response regulatory genes, including *phoU* and *phoR* decreased after degradation. Similarly, P uptake/transport genes such as *pst* and *pit* showed a declining trend. Overall, the relative abundances of both P-starvation response regulatory genes (*phoU*, *phoR*) and P uptake/transport genes (*pstABCS*, *pit*) were significantly reduced following wetland degradation (*p* < 0.05).

### 3.5 Variations in key enzymes of P-cycling metabolic pathways

**Figure 6:**
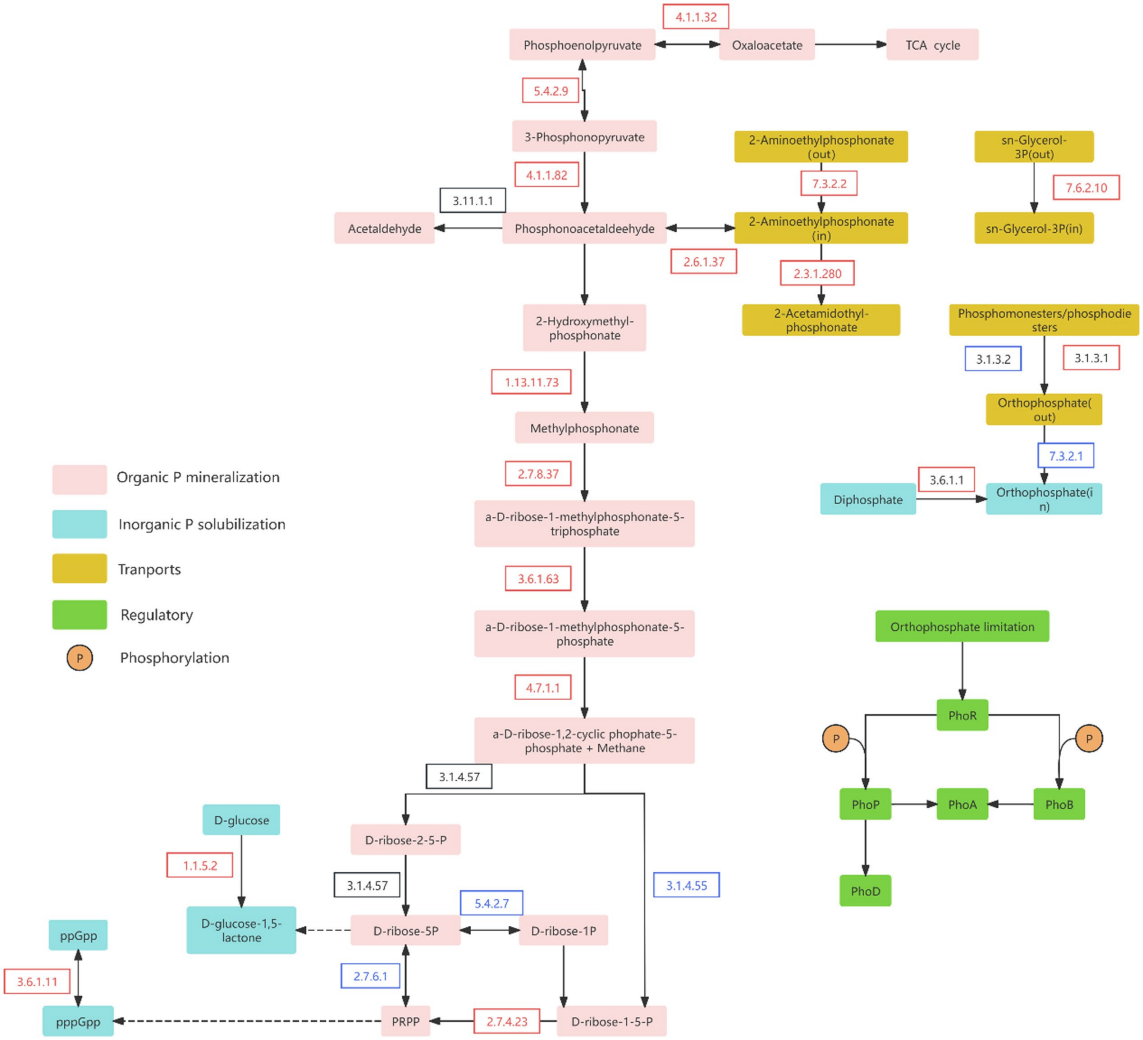
Major phosphorus metabolic pathways in soil. The boxes with different colors represent different P transformation pathways. The red and blue arrows indicate significant increases and decreases, respectively, in phosphorus cycling genes after degradation compared to non-degraded conditions. Data are reported as mean ± 1 SE (*n* = 6). Different lowercase letters in the same row mean significant difference at *p* < 0.05. PRPP, α-D-ribose-1-diphosphate-5P.

Analysis of enzymes involved in the P-cycling metabolic pathways revealed distinct changes following wetland degradation (Figure 6). Compared to ND wetlands, degraded sites showed a significant increase in the relative abundance of enzymes associated with the initial steps of the Po mineralization pathway. In contrast, the relative abundance of enzymes responsible for synthesizing PRPP in the later stages of this pathway was markedly reduced. These results suggest that although wetland degradation stimulates early-phase organic P mineralization, the final conversion to inorganic P is likely impeded by suppressed PRPP-synthesizing enzyme activity. Consequently, a considerable fraction of Po may not be fully mineralized into inorganic forms.

Simultaneously, the relative abundance of key enzymes involved in the inorganic P solubilization pathway (EC 1.1.5.2, EC 3.6.1.11, and EC 3.6.1.1) was significantly higher in degraded wetlands than in non-degraded sites (Figure 6; Supplementary Figure S2), further supporting the enhancement of inorganic P solubilization following wetland degradation. Moreover, enzymes related to P-transport and P-starvation regulation pathways showed a significantly reduced abundance in degraded wetlands, which is consistent with the trends observed in their corresponding functional genes (Figure 6; Supplementary Figure S2).

### 3.6 Pathway and factors controlling soil P fractions

**Figure 7:**
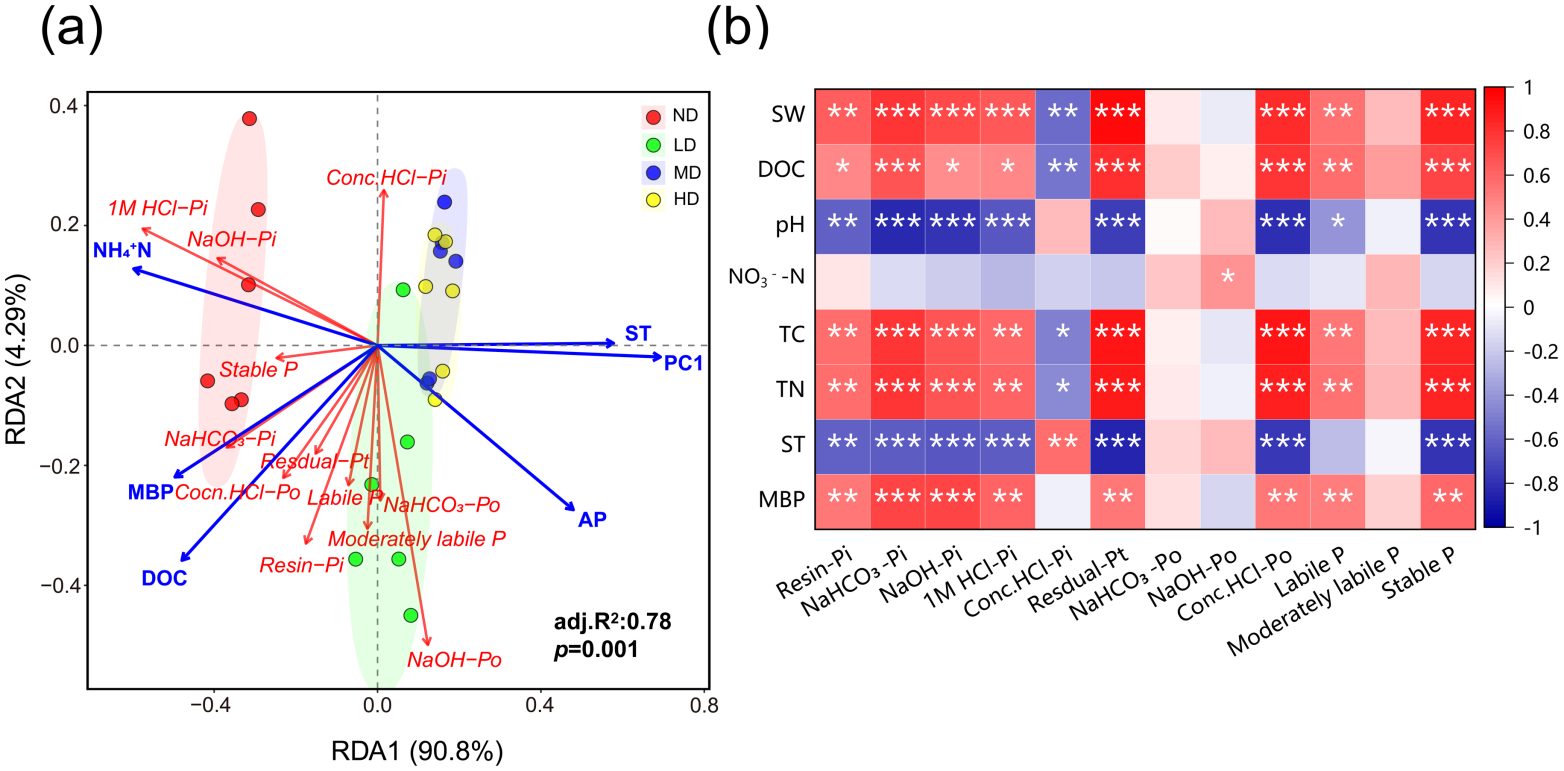
Redundancy analysis (RDA, **a**) and Spearman correlation analysis **(b)** depicting the relationships between the P fractions and selected environmental parameters in the four types of wetlands. *p < 0.05; **p < 0.01; ***p < 0.001; SW, soil water content; DOC, soil dissolved organic carbon; TC, total carbon; TN, total nitrogen; ST, soil temperature; MBP, microbial biomass phosphorus; PC1, present high-pH and low-SW. ND, non-degraded wetland; LD, slightly degraded wetland; MD, moderately degraded wetland; HD, heavily degraded wetland.

The relationship between the P factions and environmental factors was evaluated using RDA analysis (Figure 7a). Collectively, the six environmental factors explained 78% (*p* = 0.001) of the total variance in P fractions. PC1, NH_4_^+^-N, and DOC were identified as the primary influencing factors, while AP, MBP, and ST exerted secondary effects. PC1, which represents high pH and low soil water content (pH loading = 0.7, SW loading = −0.7), showed significant negative correlations with most P fractions (Figure 7; Supplementary Figures S3, S4).

**Figure 8:**
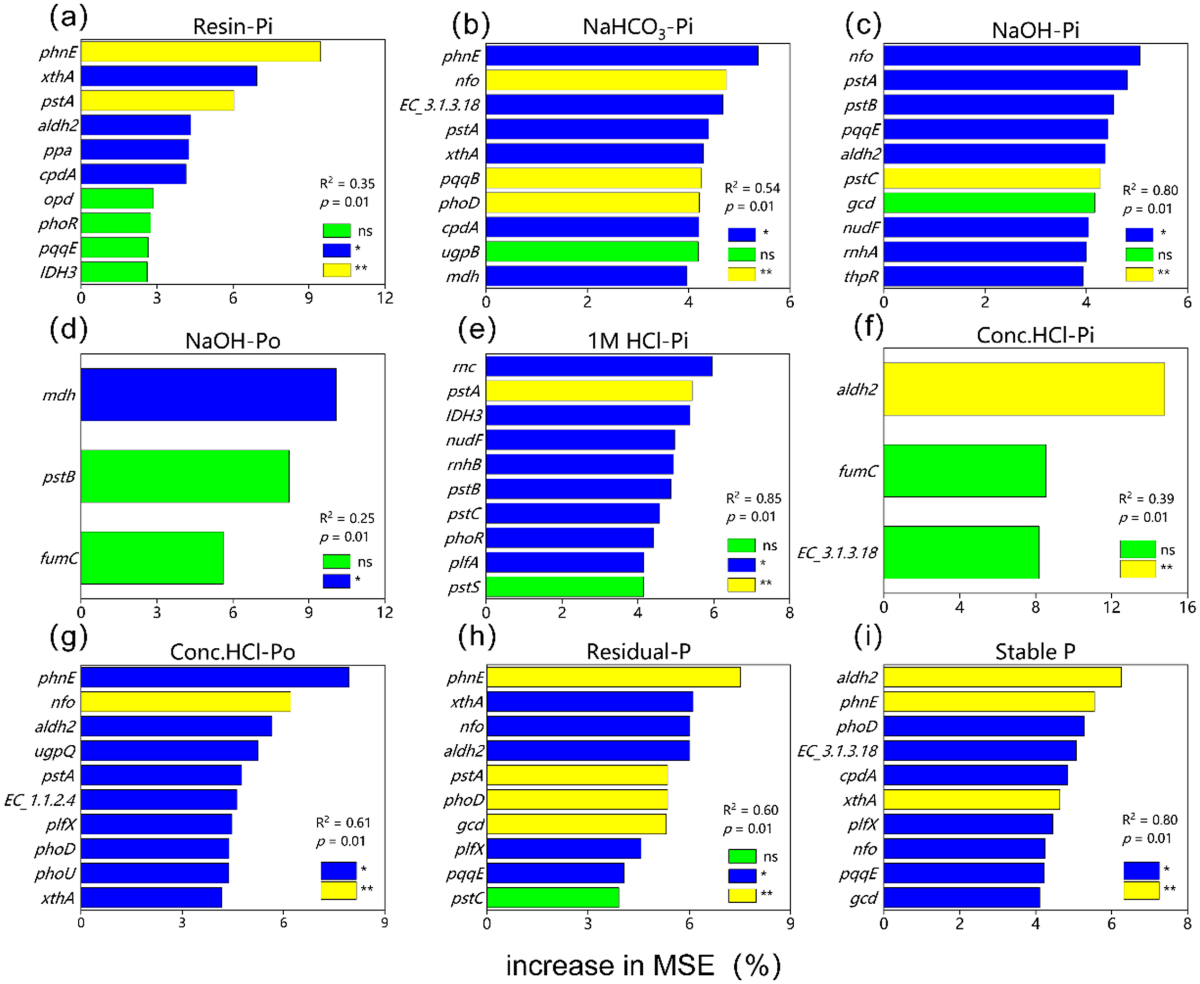
Results of mean square error (MSE, %) from a random forest aiming to identify the main driver of **(a)** Resin-Pi, **(b)** NaHCO_3_-Pi, **(c)** NaOH-Pi, **(d)** NaOH-Po, **(e)** 1 M HCl-Pi, **(f)** Conc. HCl-Pi, **(g)** Conc. HCl-Po, **(h)** Residual-P and **(i)** Stable P. *p < 0.05; **p < 0.01; ***p < 0.001, ^ns^*p* > 0.05.

The random forest analysis indicated that the genes *aldh2* and *phnE* were the major predictors of soil stable P (Figure 8). For different P fractions, *phnE*, *aldh2*, *nfo*, *rnc*, and *mdh* were identified as the main predictors for labile Pi (Conc. HCl-Pi, NaOH-Pi, 1 M HCl-Pi, and NaOH-Po, respectively).

**Figure 9:**
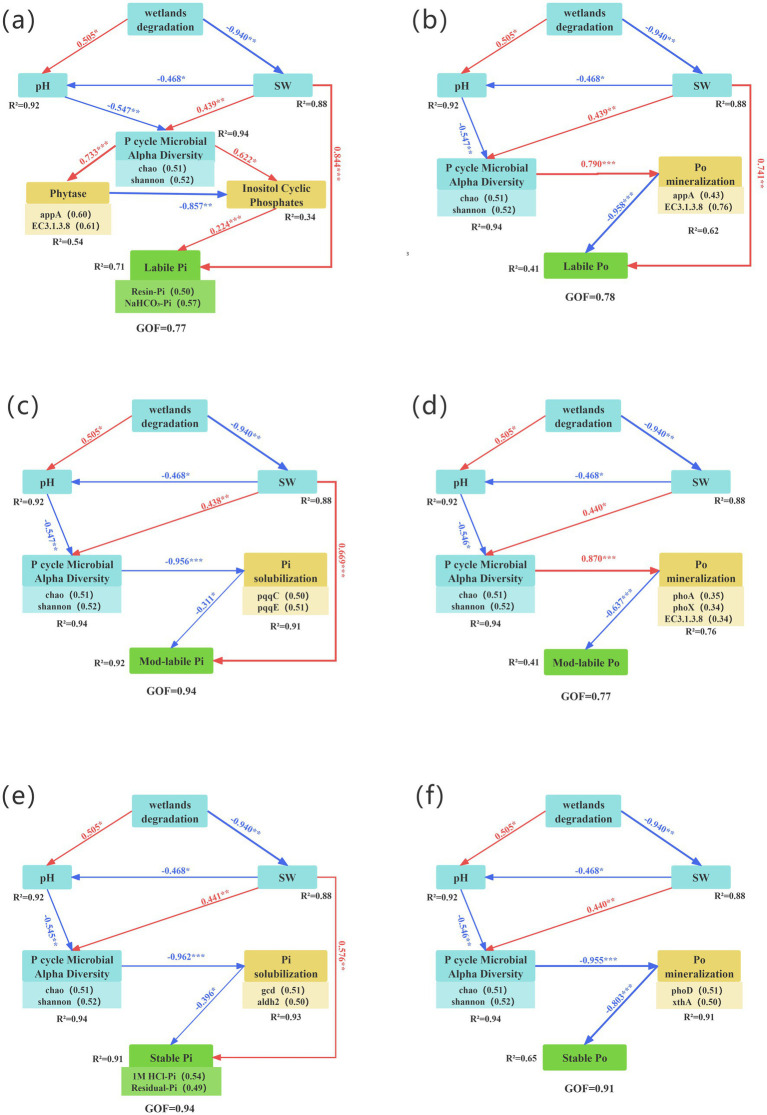
Partial least squares path modeling (PLS-PM) of the effects of wetland degradation, SW, pH, P-cycling microbial alpha diversity, and relative abundance of functional genes (involved in organic P mineralization and inorganic P solubilization) on: **(a)** labile Pi, **(b)** labile Po, **(c)** mod-labile Pi, **(d)** mod-labile Po, **(e)** stable Pi and **(f)** stable Po. The numbers above the arrows represent the size of each direct effect, and the line width is proportional to the absolute value of the direct effect. Red and blue arrows indicated positive and negative effects, respectively. Significant indicator by *p < 0.05, **p < 0.01, and ***p < 0.001. SW, soil water content. R^2^ indicates the degree to which the model explains the variance of the dependent variable. GOF is the goodness-of-fit of the model.

The partial least squares path model (PLS-PM) was used to assess the direct and indirect effects of wetland degradation, SW, pH, and P-cycling processes on various P fractions, including labile Pi, labile Po, mod-labile Pi, mod-labile Po, stable Pi, and stable Po (Figures 9a–f). Wetland degradation primarily influenced microbial diversity involved in P-cycling by altering SW and pH, which in turn affected P-cycling functional genes and P fractions. Specifically, SW exhibited strong direct positive effects on labile Pi (*r* = 0.77), labile Po (*r* = 0.78), mod-labile Pi (*r* = 0.94), and stable Pi (*r* = 0.94). Furthermore, SW exerted indirect effects on Pi fractions through microbial diversity, P-cycling genes (including Po mineralization and Pi solubilization genes), and intermediate metabolites. Both SW and pH collectively influenced Po fractions (labile Po, mod-labile Po, and stable Po) by modifying P-cycling microbial diversity and subsequently altering Po mineralization genes.

## 4 Discussion

### 4.1 Effects of wetland degradation on soil P fraction

Total soil phosphorus content gradually decreases with wetland degradation, a result that directly confirms our core hypothesis 1, which posits that wetland degradation significantly alters both the total storage and speciation of soil P. The reduction in Pi is identified as the decisive factor driving the overall decline in total phosphorus, despite an increase in Po along the degradation gradient (Figures 1a,b).

Soil P primarily originates from parent material weathering. Over long-term pedogenesis, although the proportion and content of P forms may increase, TP often decreases due to leaching (Arpiwi et al., 2012; Yang and Post, 2011). Wetland degradation is typically accompanied by drought and organic matter loss, which can promote Pi through surface runoff and changes in soil texture (Li et al., 2022; Wang et al., 2024). The reduction in moisture and rise in pH associated with degradation stimulate P-cycling microorganisms to enhance expression of Pi solubilization genes in response to environmental stress (Stirling et al., 2020), thereby accelerating dissolution processes and further reducing Pi content. Additionally, wetland degradation often leads to a shift in vegetation from hygrophytic species (e.g., *Cyperaceae*) to xerophytic grasses, which generally produce higher biomass and more litter (Hu et al., 2021). This litter constitutes a major source of Po. Due to its high carbon-to-phosphorus (C/P) ratio, it is more resistant to microbial mineralization into inorganic forms (Bai et al., 2023), thereby promoting Po accumulation and further reducing Pi. Thus, the decline in Pi—driven by degradation-induced changes in microbial activity and soil physicochemical properties—is the principal cause of the decrease in total phosphorus.

From the perspective of solubility, the proportions of labile P and mod-labile P did not change significantly along the degradation gradient, whereas stable P exhibited a continuous decline (Figure 1c). Based on solubility, soil P can be classified into labile P, moderately labile P, and stable P, each further divisible into inorganic and organic components (Hedley et al., 1982; Tiessen et al., 1984). These fractions can interconvert through solubilization, mineralization, and fixation processes, in which P-solubilizing microorganisms play a critical role (Pang et al., 2024). Labile P is the most dynamic fraction, highly sensitive to environmental changes and subject to rapid depletion or immobilization (Dierberg et al., 2021; Jiang et al., 2024; Qin et al., 2023). Although the total labile P pool remains relatively stable after wetland degradation, its compositional shifts markedly: labile Pi (Resin-P and NaHCO_3_-Pi) decreases significantly, while labile Po (NaHCO_3_-Po) increases (Figures 2d,f). Previous studies indicate that labile Pi stability is strongly influenced by SW and pH, with wetland degradation-induced drought and elevated pH potentially reducing its concentration (Zhang et al., 2020). On the one hand, reduced vegetation cover and weakened hydrological regulation after degradation intensify runoff erosion, promoting labile Pi loss (Pollock and Norman, 2025). On the other hand, the decomposition of dead microorganisms and plants returns organic phosphorus to the soil, increasing labile Po content (Jiang et al., 2024; Li et al., 2024; Yang et al., 2014).

Moderately labile P serves as a key transitional pool in soil P transformation, bridging stable P solubilization and labile P fixation (Liu et al., 2021b; Tiessen et al., 1984). Following degradation, moderately labile P initially increases then decreases, with moderately labile Pi (NaOH-Pi) declining significantly and moderately labile Po (NaOH-Po) showing an increasing trend (Figures 2d,e). First, wetland degradation alters the composition of soil P-cycling microbial communities, enriching P-solubilizing microorganisms (Li et al., 2022), which enhances Pi solubilization and Po mineralization, thereby reducing moderately labile Pi. Secondly, changes in soil physicochemical properties affect ion exchange processes, reducing adsorption sites for moderately labile Pi and further diminishing its content (Jiang et al., 2024). The increase in moderately labile Po may stem from enhanced mineralization of stable Po, as Po undergoes progressive degradation from stable to labile forms (Qin et al., 2023). Our data show a significant decrease in stable Po in degraded wetlands (Figure 2), supporting the transformation of stable Po into moderately labile and labile Po, indicating a shift from stable to more labile phosphorus pools.

Stable P, being the most resistant to loss, typically requires solubilization or mineralization into moderately labile P before further transformation (Hedley et al., 1982; Tiessen et al., 1984). Wetland degradation reduces vegetation cover, increasing soil susceptibility to weathering and erosion (Tang et al., 2021). In mildly weathered soils, the observed decline in stable P may result from its conversion to moderately labile P, acting as a buffer for AP (Guo et al., 2000). Moreover, degradation-induced shifts in microbial community structure significantly influence P solubilization and mineralization processes. Global studies indicate that grassland ecosystems harbor higher abundances of Pi solubilization and Po mineralization genes than wetlands (Wang et al., 2024). Thus, the conversion of wetlands to grasslands may enhance these microbial functions, ultimately reducing stable P content (Li et al., 2022).

In conclusion, wetland degradation significantly alters soil P fractions by affecting both physical processes (erosion and weathering) and biological processes (microbial-mediated transformations), collectively reshaping P cycling in degraded wetland ecosystems.

### 4.2 Effects of wetland degradation on soil physicochemical properties

Wetland degradation leads to pronounced changes in soil moisture and pH, which drive an increase in soil AP content, albeit accompanied by reductions in microbial P, carbon, and nitrogen pools (Figure 3). During this process, soil water content is often the first parameter to be affected. Most studies indicate that wetland degradation results in a significant decline in soil moisture, which in turn alters the forms and distribution of soil P (Wu et al., 2021).

In addition to reducing soil moisture, wetland degradation also elevates soil pH. The decrease in water content typically improves soil aeration and promotes the decomposition of organic acids, ultimately leading to increased pH levels (Wang et al., 2015). This study further demonstrates that dissolved organic carbon, total nitrogen, and ammonium nitrogen contents decrease significantly with increasing degradation severity. On one hand, degradation reduces vegetation cover and increases surface exposure, accelerating the loss of topsoil nutrients through wind and water erosion (Arroyo et al., 2015). On the other hand, it diminishes plant productivity, aboveground biomass, and litterfall, thereby reducing organic matter inputs and weakening the soil’s nutrient supply (Yang et al., 2021).

In summary, key soil physicochemical properties respond distinctly to wetland degradation. The process induces substantial reductions in soil moisture and organic matter, coupled with elevated pH, collectively impairing the health and stability of wetland ecosystems.

### 4.3 Effects of wetland degradation on soil P-cycling microbial diversity and functional genes

Wetland degradation reduces the diversity of microorganisms involved in P cycling, providing direct support for the core postulate of hypothesis 2, which states that degradation significantly restructures the soil microbial community. Soil water content strongly influences microbial activity and composition, thereby playing a critical role in regulating soil P cycling (Xu et al., 2023). Studies indicate that degraded wetlands exhibit significantly lower SW than non-degraded wetlands, accompanied by a notable reduction in the alpha diversity (Shannon and Chao1 indices) of P-cycling microorganisms (Supplementary Figure S1). Under drought conditions, soil microbial communities shift toward aerobic taxa and become increasingly adapted to water stress as moisture declines (Chaves et al., 2003; Manzoni et al., 2014). In addition to SW, the rise in pH commonly associated with wetland degradation also alters microbial community structure. Elevated pH can eliminate acidophilic P-cycling microorganisms (e.g., *Acidophilus*) while favoring the proliferation of other P-cycling taxa (Huang et al., 2017; Liang et al., 2020). Since these microorganisms are key hosts of P-cycling functional genes, such compositional shifts directly affect P transformation processes in the soil.

Compared with non-degraded wetlands, degraded sites showed a significant increase the relative abundance of most functional genes related to phosphorus mineralization (Po mineralization) and phosphorus solubilization (Pi solubilization), while genes associated with P-starvation response regulation and P uptake/transport were significantly decreased (Figure 5). These results align with previous reports indicating that wetland degradation first alters SW and pH, which in turn affect microbial diversity, and ultimately reshape the functional genes potential for P cycling (Li et al., 2022; Wang et al., 2024). The decline in SW and increased oxygen diffusion following degradation promote the activity of aerobic microorganisms (e.g., *Proteobacteria* and *Firmicutes*), which are major carriers of Po mineralization and Pi solubilization genes (e.g., *phoD*, *gcd*, *ppx*, and *ppa* genes) (Pang et al., 2024), thereby explaining the increased abundance of these genes. Concurrently, reduced SW induces oxidative stress under arid conditions, likely causing microorganisms to divert energy toward stress adaptation (e.g., synthesizing antioxidant enzymes) rather than P uptake and transport (Bista et al., 2018; Li et al., 2023), resulting in the downregulation of genes such as *pstABS* and *pit*. Furthermore, the P-starvation response is known to be regulated by soil P availability; higher P levels suppress the expression of two-component regulatory genes such as *phoR* and *phoB* (Rawat et al., 2020). The increase in AP observed in this study further supports this mechanism.

Notably, although the overall relative abundance of Po mineralization genes increased, analysis of individual gene abundances and metabolic pathways revealed a decline in terminal mineralization genes and enzymes (e.g., *phoA*, *appA*). While this seems inconsistent with hypothesis 2 and the general trend in organic phosphorus mineralization gene abundance, these terminal genes and enzymes are crucial for determining whether organic P is fully mineralized into inorganic forms (Pang et al., 2024). Soil bacteria generally prefer environments with higher organic carbon content (Lehmann et al., 2017; Wang et al., 2016), and P-solubilizing bacteria are particularly sensitive to environmental factors such as soil organic carbon (Ragot et al., 2015). Low total organic carbon (TOC) —especially dissolved organic carbon (DOC)—reduces the diversity and activity of P-solubilizing bacteria (Luo et al., 2017). Po mineralization and alkaline phosphatase secretion are energy-intensive processes that require substantial carbon investment (Nannipieri et al., 2011). Following wetland degradation, the reduction in soil organic carbon forces microorganisms to allocate limited carbon resources toward basic growth and maintenance rather than Po mineralization (Alhassan et al., 2018; Ma W. et al., 2020; Wu et al., 2020). Thus, carbon limitation likely underlies the decreased abundance of terminal Po mineralization genes.

### 4.4 Mechanism of P fractions response to wetland degradation

**Figure 10:**
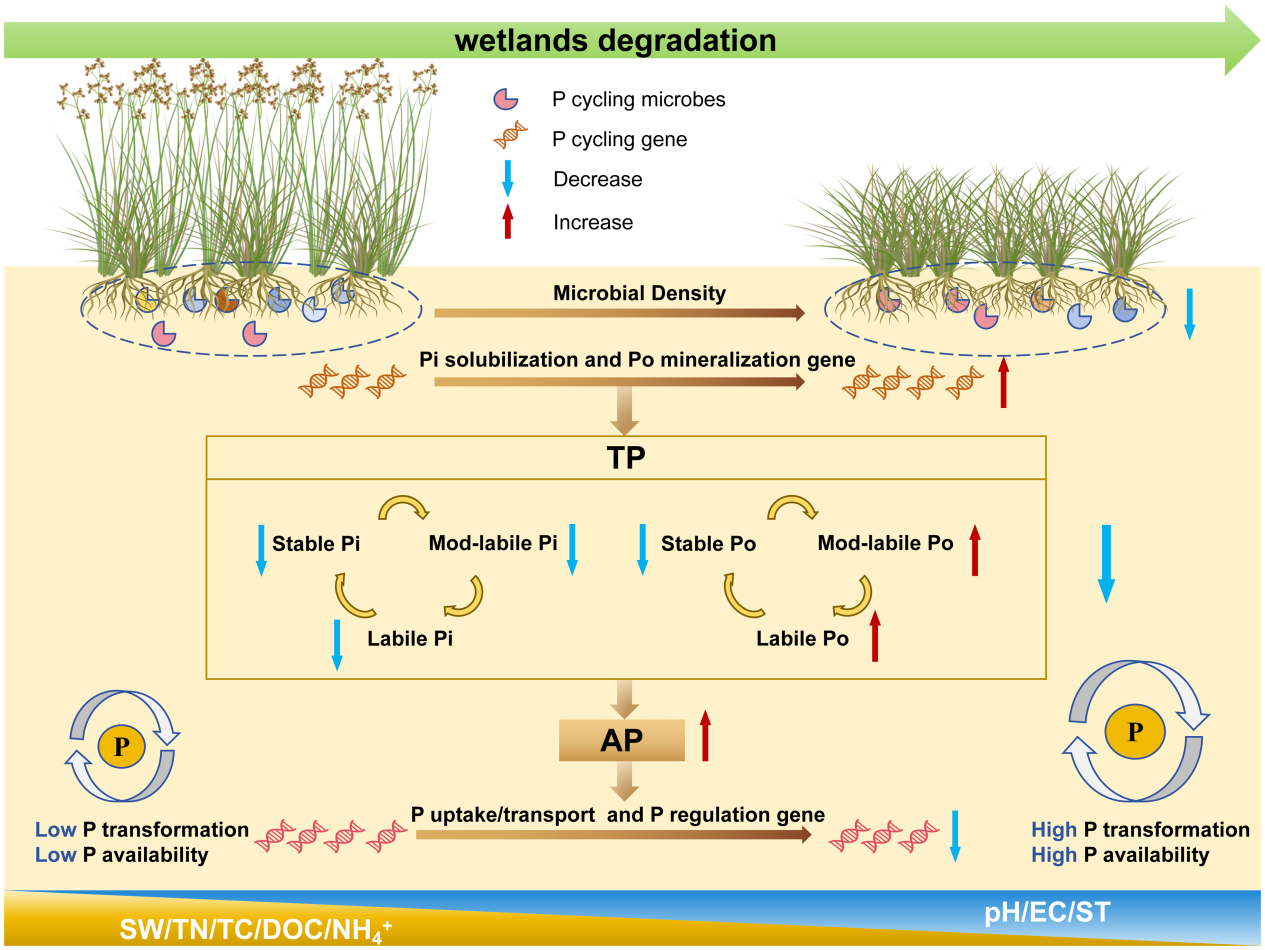
Conceptual diagram of the effects of wetland degradation on soil phosphorus fractions and phosphorus availability. EC, electrical conductivity; ST, soil temperature; DOC, dissolved organic carbon; TC, total carbon; TN, total nitrogen.

Overall, wetland degradation influences the diversity of P-cycling microorganisms by modulating SW and pH, which subsequently alters the abundance of specific P-cycling functional genes and ultimately affects the dynamics of labile P, mod-labile P, and stable P pools (Figures 9, 10). This chain-reaction mechanism of “environmental factors → microbial community → functional genes → phosphorus transformation” provides comprehensive validation for hypothesis 3 proposed in this study.

Soil P assimilation capacity is largely governed by the abundance of P-cycling genes (e.g., *pqqBCDE*, *gcd*, *appA*, *phoD*, and *phoA*), which are in turn regulated by soil environmental conditions (Dai et al., 2020; Hsieh and Wanner, 2010; Rasul et al., 2019; Rawat et al., 2020). Recent studies indicate that SW, pH, microbial C: P, AP, and DOC significantly influence soil P transformation (Dai et al., 2020; Spohn and Kuzyakov, 2013; Wang et al., 2015). The partial least squares structural equation modeling (PLS-SEM) clearly delineates this causal pathway: wetland degradation exerts a decisive influence on P cycling processes by reducing soil water content and increasing pH. This aligns with observations from marsh-to-meadow degradation, where declining groundwater levels cause upward movement of salts and alkaloids, elevating topsoil pH and subsequently altering soil P cycling (Li et al., 2022).

First, wetland degradation reduces the diversity of P-cycling microorganisms via changes in SW and pH, leading to a decline in key Po mineralization genes (*appA*, *EC3.1.4.55*). This results in the accumulation of labile Po (e.g., inositol cyclic phosphate) and limited availability of organic sources of labile Pi (Figures 9a,b). Inositol phosphates (e.g., phytate) constitute a major fraction of soil Po (Lu et al., 2020). The cyclic form of inositol phosphate serves both as a substrate for Po mineralization and an intermediate in its degradation (Connolly et al., 1986). Although Po mineralization typically reduces inositol phosphate and its metabolites (Zhou et al., 2019), the decreased abundance of key enzymes (e.g., phytase, EC 3.1.4.55, and EC 2.7.6.1) following degradation impedes direct conversion to Pi. Instead, these compounds accumulate as readily mineralizable Po forms (NaHCO₃-Po and NaOH-Po) (Zhou et al., 2019). Consequently, although inositol cyclic phosphate contributes slightly to labile Pi (path coefficient = 0.224), its effect is constrained by the reduced expression of mineralization-associated genes (Figure 9a).

Second, wetland degradation-induced shifts in SW and pH reduce microbial diversity, which nevertheless promotes an increase in the relative abundance of Pi solubilization genes (e.g., *pqqCE*, *gcd*, and *aldh2*) and certain Po mineralization genes (such as *phoD* and *xthA*). This gene enrichment ultimately leads to reductions in moderately labile Pi, stable Pi, and stable Po (Figures 9c,e,f). Grassland ecosystems, which often succeed degraded wetlands, host higher abundances of Pi solubilization and Po mineralization genes than wetlands, providing a more favorable environment for P-solubilizing microorganisms (Wang et al., 2024). The loss of microbial diversity and reduced interspecific competition during degradation allow these taxa to dominate, increasing the abundance of genes involved in Pi solubilization and Po mineralization (Liu et al., 2023; Wang et al., 2024). Consistent with previous studies (Li et al., 2022; Liu et al., 2023; Zeng et al., 2022; Zhang et al., 2025), this increase facilitates the conversion of moderately labile Pi, stable Pi, and stable Po into AP, enhancing soil P availability and confirming the transformation of forms as postulated in hypothesis 1.

Partial least squares structural equation modeling further indicates that reduced SW directly affects the concentrations of labile Pi, labile Po, moderately labile Pi, and stable Pi. This is consistent with earlier findings that moisture decline reduces Pi in the most bioavailable pools and suppresses phosphatase activity, thereby limiting Po mineralization and further decreasing labile Pi (Zhang et al., 2020). In summary, the mechanisms through which wetland degradation affects various P fractions are broadly consistent: degradation primarily regulates P-cycling microbial diversity through SW and pH, thereby modifying the abundance of P-cycling functional genes and ultimately reshaping soil phosphorus composition (Figure 10).

## 5 Conclusion

The findings of this study offer valuable insights into the complex effects of wetland degradation on soil P fractions in temperate wetland ecosystems. Our results demonstrate that total soil phosphorus content decreases with increasing degradation intensity, primarily driven by a reduction in Pi. From the perspective of P solubility, wetland degradation facilitates the interconversion among labile P, mod-labile P, and stable P. Specifically, wetland degradation modulates the diversity of functionally relevant microorganisms via changes in soil moisture and pH, promoting the transformation of stable P into mod-labile P and subsequently into labile P. It is noteworthy that this study found a reduction in terminal functional genes and enzymes involved in the Po mineralization pathway following wetland degradation, which impedes the complete mineralization process and leads to the accumulation of stable Po degraded into labile Po. As a result, Po content increases with degradation intensity. Water loss and elevated pH during wetland degradation significantly reduce Pi content. On the one hand, these altered environmental conditions limit the mineralization of Po, while on the other hand, runoff during rainfall events further contributes to Pi loss. The degradation process is initially characterized by a decline in soil moisture, which regulates pH and other physicochemical properties. These changes further drive shifts in microbial community diversity, influence the abundance of P-cycling functional genes, and modulate P metabolic processes, ultimately altering the forms and total pool of soil phosphorus. Collectively, P loss during wetland degradation not only disrupts the ecological balance of the wetland ecosystem itself but also contributes to pollution in adjacent river systems and surrounding environments. Therefore, nutrient losses associated with wetland degradation warrant serious attention and mitigation efforts.

## Data Availability

The original contributions presented in the study are publicly available. This data can be found here: https://doi.org/10.6084/m9.figshare.30586556.v2.

## Footnotes

^1^ http://www.illumina.com

^2^ http://www.majorbio.com

^3^https://github.com/voutcn/megahit, version 1.1.2.

^4^https://github.com/hyattpd/Prodigal, version 2.6.3.

^5^http://weizhongli-lab.org/cd-hit/, version 4.7.

^6^https://github.com/ShujiaHuang/SOAPaligner, version soap2.21release.

^7^https://github.com/bbuchfink/diamond, version 2.0.13.
