# Supplementary material for: Wetland degradation promotes soil P fraction transformation by altering P-cycling functional genes and metabolic pathways
Source: Front Microbiol. 2025 Nov 12;16:1677320. doi: 10.3389/fmicb.2025.1677320 (PMC12648965; doi:10.3389/fmicb.2025.1677320)
Supplement: Supplementary file 2 [file Supplementary_file_1.docx]

**Wetland degradation promotes soil P fraction transformation by altering P-cycling functional genes and metabolic pathways**

Yumeng Jiang^a^, Yu Zou^a^, Miaojia Sun^a^, Weihong Zhu^a,^*, Wanling Xu^a,^*

^a^ College of Geography and Ocean Sciences, Yanbian University, Hunchun 133300, China

*Author for correspondence

**Wanling Xu, Weihong Zhu**

College of Geography and Ocean Sciences

Yanbian University

Hunchun Jilin Province, P. R. China 133300

Tel.: +86 433 8175148

E-mail address: [wlxu517@ybu.edu.cn](mailto:wlxu517@ybu.edu.cn) (W. Xu); [whzhu@ybu.edu.cn](mailto:whzhu@ybu.edu.cn) (W. Zhu)

## **Supplementary information**

## **Table S1.**

The soil biogeochemical properties in the four treatments. Data are reported as mean ± 1 SE (n = 6). Different lowercase letters in the same row mean significant difference at *P* < 0.05 among the four treatments. SW, soil water content; TP, total phosphorus; MBP, microbial biomass phosphorus; AP, available phosphorus; TC, total carbon; TN, total nitrogen; ND, non-degraded wetland; LD, slightly degraded wetland; MD, moderately degraded wetland; HD, heavily degraded wetland.

| Soil properties | ND | LD | MD | HD |
| --- | --- | --- | --- | --- |
| pH | 5.05 ± 0.89 c | 5.50 ± 0.07 b | 5.64 ± 0.07a | 5.57 ± 0.02 ab |
| SW | 337.67 ± 97.30 a | 59.37 ± 15.21 ab | 43.72 ± 3.06 bc | 34.15 ± 1.85 c |
| TP | 732.58 ± 183.43 a | 602.40 ± 103.47 ab | 517.38 ± 25.70 ab | 495.57 ± 21.67 b |
| MBP | 43.72 ± 15.53 a | 25.31 ± 6.57 ab | 15.90 ± 4.96 b | 25.82 ± 13.23 ab |
| AP | 2.05 ± 0.48 b | 2.64 ± 0.27 a | 2.77 ± 0.24 a | 2.91 ± 0.24 a |
| TC | 154.13 ± 18.25 a | 48.19 ± 12 b | 34.84 ± 2.58b | 30.46 ± 2.51 b |
| TN | 10.55 ± 1.51 a | 4.54 ± 0.89 b | 3.57 ± 0.25 b | 3.36 ± 0.20 b |
| NH_4_^+^N | 42.83 ± 26.77 a | 11.76 ± 2.71 b | 12.78 ± 2.59 b | 12.39 ± 2.91 b |
| NO_3_^-^N | 0.85 ± 0.41 c | 3.79 ± 0.98 a | 1.22 ± 0.11 c | 2.51 ± 0.34 b |

## **Table S2.**

The average concentration of soil P fractions (mg kg^-1^) in different treatments. Data are reported as mean ± 1 SE (n = 6). Different lowercase letters in the same row mean significant difference at *P* < 0.05 among the four treatments. ND, non-degraded wetland; LD, slightly degraded wetland; MD, moderately degraded wetland; HD, heavily degraded wetland.

| P fraction | ND | | LD | MD | HD |
| --- | --- | --- | --- | --- | --- |
| ***Labile P*** |  | |  |  |  |
| Resin-Pi | 0.73 ± 0.29 a | | 0.69 ± 0.19 a | 0.36 ± 0.08 b | 0.38 ± 0.09 b |
| NaHCO_3_-Pi | 17.60 ± 6.73 a | | 7.89 ± 1.42 a | 4.46 ± 0.45 b | 5.83 ± 0.9 b |
| NaHCO_3_-Po | 36.24 ± 8.96 a | | 41.11 ± 8.34 a | 39.19 ± 2.85a | 37.27 ± 2.90 a |
| ∑Labile P | 54.57 ± 15.01 a | | 49.68 ± 9.59 a | 44.01 ± 3.02 a | 43.48 ± 3.45 a |
| ***Moderately labile P*** |  | |  |  |  |
| NaOH-Pi | 167.08 ± 39.45 a | | 61.32 ± 12.66 b | 42.02 ± 7.21 b | 51.98 ± 7.99 b |
| NaOH-Po | 160.50 ± 86.80 a | | 290.52 ± 60.77 a | 276.10 ± 21.54 a | 244.97 ± 11.35 a |
| ∑Moderately labile P | 327.57 ± 118.16 a | | 351.84 ± 69.45 a | 318.12 ± 19.54 a | 296.95 ± 14.54 a |
| ***Stable P*** |  | |  |  |  |
| 1M HCl-Pi | 140.34 ± 27.78 a | | 37.18 ± 20.69 b | 21.54 ± 8.90 b | 22.10 ± 10.98 b |
| Conc.HCl-Pi | 34.14 ± 2.44 ab | | 26.26 ± 1.44 b | 34.84 ± 2.04 ab | 39.86 ± 1.56 a |
| Conc.HCl-Po | 52.71 ± 14.77 a | | 38.13 ± 7.76 a | 23.98 ± 2.21 b | 22.32 ± 4.41 b |
| Resdual-Pt | 123.24 ± 20.21 a | | 99.31 ± 6.90 a | 74.9 ± 2.31 b | 70.87 ± 3.14 b |
| ∑Stable P | 350.44 ± 56.37 a | | 200.87 ± 30.99 b | 155.25 ± 7.64 b | 155.14 ± 14.26 b |
| Total Pi | 483.13 ± 86.30 a | | 232.64 ± 36.05 b | 178.11 ± 11.48 b | 191.01 ± 17.04 b |
| Total Po | | 249.45 ± 101.91 a | 369.76 ± 74.57 a | 339.27 ± 24.22 a | 304.56 ± 11.60 a |

## **Table S3.**

KO number, function, gene name and P cycling functional classification of the investigated genes referring to the KEGG database.

| P cycling functions | KO number | Gene name | Gene function | Function group |
| --- | --- | --- | --- | --- |
| Inorganic P solubilization | K01507 | *ppa* | inorganic pyrophosphatase | inorganic pyrophosphatase |
|  | K00117 | *gcd* | pyruvate ferredoxin oxidoreductase beta subunit, gluconic acid | PQQ-GDH |
|  | K06135 | *pqqA* | pyrroloquinoline quinone biosynthesis protein A, gluconic acid | PQQ-GDH |
|  | K06136 | *pqqB* | pyrroloquinoline quinone biosynthesis protein B, gluconic acid | PQQ-GDH |
|  | K06137 | *pqqC* | pyrroloquinoline-quinone synthase, gluconic acid | PQQ-GDH |
|  | K06138 | *pqqD* | pyrroloquinoline quinone biosynthesis protein D, gluconic acid | PQQ-GDH |
|  | K06139 | *pqqE* | PqqA peptide cyclase, gluconic acid | PQQ-GDH |
|  | K08355 | *aoxA* | arsenite oxidase small subunit, oxalic acid | organic acid |
|  | K00016 | *ldh* | L-lactate dehydrogenase, lactic acid | organic acid |
|  | K00102 | *EC:1.1.2.4* | putative D-lactate dehydrogenase, lactic acid | organic acid |
|  | K00128 | *aldh2* | aldehyde dehydrogenase, acetic acid | organic acid |
|  | K04069 | *plfA* | pyruvate formate lyase activating enzyme, formic acid | organic acid |
|  | K04070 | *plfX* | putative pyruvate formate lyase activating enzyme, formic acid | organic acid |
|  | K00656 | *plfB* | pyruvate formate lyase, formic acid | organic acid |
|  | K00122 | *FDH* | formate dehydrogenase, formic acid | organic acid |
|  | K01455 | *E3.5.1.49* | formamidase, formic acid | organic acid |
|  | K03779 | *ttdA* | L (+)-tartrate dehydratase alpha subunit, tartaric acid | organic acid |
|  | K03780 | *ttdB* | L (+)-tartrate dehydratase beta subunit, tartaric acid | organic acid |
|  | K00024 | *mdh* | malate dehydrogenase, malic acid | organic acid |
|  | K01679 | *fumC* | fumarate hydratase, fumaric acid | organic acid |
|  | K00030 | *IDH3* | isocitrate dehydrogenase (NAD+), Isocitric acid | organic acid |
| Organic P mineralization | K05774 | *phnN* | C-P lyase subunit, ribose 1,5-bisphosphokinase | C-P relevant |
|  | K05780 | *phnL* | C-P lyase subunit, alpha-D-ribose 1-methylphosphonate 5-triphosphate synthase | C-P relevant |
|  | K05781 | *phnK* | C-P lyase subunit, alpha-D-ribose 1-methylphosphonate 5-triphosphate synthase | C-P relevant |
|  | K09994 | *phnO* | C-P lyase subunit, aminoalkylphosphonate N-acetyltransferase | C-P relevant |
|  | K06162 | *phnM* | C-P lyase subunit, alpha-D-ribose 1-methylphosphonate 5-triphosphate diphosphatase | C-P relevant |
|  | K06163 | *phnJ* | C-P lyase subunit, alpha-D-ribose 1-methylphosphonate 5-phosphate C-P lyase | C-P relevant |
|  | K06164 | *phnI* | C-P lyase subunit, alpha-D-ribose 1-methylphosphonate 5-triphosphate synthase | C-P relevant |
|  | K06165 | *phnH* | C-P lyase subunit, alpha-D-ribose 1-methylphosphonate 5-triphosphate synthase | C-P relevant |
|  | K06166 | *phnG* | C-P lyase subunit, alpha-D-ribose 1-methylphosphonate 5-triphosphate synthase | C-P relevant |
|  | K06167 | *phnP* | C-P lyase subunit, phosphoribosyl 1,2-cyclic phosphate phosphodiesterase | C-P relevant |
|  | K02043 | *phnF* | C-P lyase subunit, GntR family transcriptional regulator, phosphonate transport system regulatory protein | C-P relevant |
|  | K06193 | *phnA* | phosphonoacetate hydrolase | C-P relevant |
|  | K05306 | *phnX* | phosphonoacetaldehyde hydrolase | C-P relevant |
|  | K03430 | *phnW* | 2-aminoethylphosphonate-pyruvate transaminase | C-P relevant |
|  | K01093 | *appA* | 4-phytase / acid phosphatase | phosphomonoesterase |
|  | K01083 | *E3.1.3.8* | 3-phytase | phosphomonoesterase |
|  | K01078 | *olpAPHO* | acid phosphatase | phosphomonoesterase |
|  | K09474 | *phoN* | acid phosphatase (class A) | phosphomonoesterase |
|  | K03788 | *aphA* | acid phosphatase (class B) | phosphomonoesterase |
|  | K01077 | *phoA* | alkaline phosphatase | phosphomonoesterase |
|  | K01113 | *phoD* | alkaline phosphatase | phosphomonoesterase |
|  | K07093 | *phoX* | alkaline phosphatase | phosphomonoesterase |
|  | K01092 | *suhB* | myo-inositol-1(or 4)-monophosphatase | phosphomonoesterase |
|  | K19504 | *gfrE* | glucoselysine-6-phosphate deglycase | phosphomonoesterase |
|  | K00036 | *G6PD* | glucose-6-phosphate 1-dehydrogenase | phosphomonoesterase |
|  | K18978 | *gapN* | glyceraldehyde-3-phosphate dehydrogenase [NAD(P)+] | phosphomonoesterase |
|  | K00057 | *gpsA* | glycerol-3-phosphate dehydrogenase (NAD(P)+) | phosphomonoesterase |
|  | K00115 | *gld* | glucose 1-dehydrogenase | phosphomonoesterase |
|  | K01095 | *pgpA* | phosphatidylglycerophosphatase A | phosphomonoesterase |
|  | K01097 | *pgpC* | phosphatidylglycerophosphatase C | phosphomonoesterase |
|  | K01513 | *ENPP1_3* | ectonucleotide pyrophosphatase/phosphodiesterase family member | phosphodiesterase |
|  | K08726 | *EPHX2* | soluble epoxide hydrolase / lipid-phosphate phosphatase | phosphodiesterase |
|  | K01114 | *plc* | phospholipase C | phosphodiesterase |
|  | K01115 | *PLD1_2* | phospholipase D1/2 | phosphodiesterase |
|  | K01124 | *GDPD2* | glycerophosphoinositol inositolphosphodiesterase | phosphodiesterase |
|  | K03651 | *cpdA* | 3',5'-cyclic-AMP phosphodiesterase | phosphodiesterase |
|  | K01975 | *thpR* | RNA 2',3'-cyclic 3'-phosphodiesterase | phosphodiesterase |
|  | K06167 | *phnP* | phosphoribosyl 1,2-cyclic phosphate phosphodiesterase | phosphodiesterase |
|  | K08722 | *yfbR* | 5'-deoxynucleotidase | phosphodiesterase |
|  | K03787 | *surE* | 5'-nucleotidase | phosphodiesterase |
|  | K01141 | *sbcB* | exodeoxyribonuclease I | phosphodiesterase |
|  | K01142 | *xthA* | exodeoxyribonuclease III | phosphodiesterase |
|  | K01150 | *endA* | deoxyribonuclease I | phosphodiesterase |
|  | K01151 | *nfo* | deoxyribonuclease IV | phosphodiesterase |
|  | K05982 | *nfi* | deoxyribonuclease V | phosphodiesterase |
|  | K01158 | *DNASE2* | deoxyribonuclease II | phosphodiesterase |
|  | K03685 | *rnc* | ribonuclease III | phosphodiesterase |
|  | K03469 | *rnhA* | ribonuclease HI | phosphodiesterase |
|  | K03470 | *rnhB* | ribonuclease HII | phosphodiesterase |
|  | K03471 | *rnhC* | ribonuclease HIII | phosphodiesterase |
|  | K03536 | *rnpA* | ribonuclease P protein component | phosphodiesterase |
|  | K08300 | *rne* | ribonuclease E | phosphodiesterase |
|  | K18105 | *rtcA* | RNA 3'-terminal phosphate cyclase (GTP) | phosphodiesterase |
|  | K01126 | *ugpQ* | glycerophosphoryl diester phosphodiesterase | phosphodiesterase |
|  | K18916 | *ptxD* | phosphonate dehydrogenase | phosphonatase |
|  | K01091 | *EC:3.1.3.18* | phosphonatase-like hydrolase, phosphoglycolate phosphatase | phosphonatase |
|  | K01524 | *ppx* | exopolyphosphatase / guanosine-5'-triphosphate,3'-diphosphate pyrophosphatase | organic pyrophosphatase |
|  | K01515 | *nudF* | ADP-ribose pyrophosphatase | organic pyrophosphatase |
|  | K07048 | *opd* | phosphotriesterase | phosphotriesterase |
| P starvation response regulation | K02039 | *phoU* | phoR/phoB inhibitor protein phoU |  |
|  | K07636 | *phoR* | two-component system, OmpR family, phosphate regulon sensor histidine kinase PhoR |  |
|  | K07657 | *phoB* | two-component system, OmpR family, phosphate regulon response regulator PhoB |  |
|  | K07659 | *ompR* | two-component system, OmpR family, phosphate regulon response regulator OmpR |  |
|  | K06140 | *rnk* | phosphate system positive regulatory protein PHO4 |  |
|  | K02444 | *glpR* | DeoR family transcriptional regulator, glycerol-3-phosphate regulon repressor |  |
| P uptake and transport system | K02036 | *pstB* | phosphate transport system, ATP-binding component, phosphate transport system ATP-binding protein | phosphate transport system |
|  | K02037 | *pstC* | phosphate transport system, membrane component, phosphate transport system permease protein | phosphate transport system |
|  | K02038 | *pstA* | phosphate transport system, membrane component, phosphate transport system permease protein | phosphate transport system |
|  | K02040 | *pstS* | phosphate transport system, periplasmic-binding component, phosphate transport system substrate-binding protein | phosphate transport system |
|  | K03306 | *pit* | inorganic phosphate transporter | phosphate transport system |
|  | K08176 | *PHO84* | inorganic phosphate transporter | phosphate transport system |
|  | K08176 | *PHT1* | inorganic phosphate transporter | phosphate transport system |
|  | K05813 | *ugpB* | Glycerol-3-phosphate transporter subunit, periplasmic-binding component | GP phosphate transport system |
|  | K05814 | *ugpA* | Glycerol-3-phosphate transporter subunit | GP phosphate transport system |
|  | K05815 | *ugpE* | Glycerol-3-phosphate transporter subunit, membrane component | GP phosphate transport system |
|  | K05816 | *ugpC* | Glycerol-3-phosphate transporter subunit, ATP-binding component | GP phosphate transport system |
|  | K17240 | *inoK* | inositol-phosphate transport system ATP-binding protein | IP phosphate transport system |
|  | K17237 | *inoE* | inositol-phosphate transport system substrate-binding protein | IP phosphate transport system |
|  | K17238 | *inoF* | inositol-phosphate transport system permease protein | IP phosphate transport system |
|  | K17239 | *inoG* | inositol-phosphate transport system permease protein | IP phosphate transport system |
|  | K02041 | *phnC* | phosphonate transport system, ATP-binding component | phosphonate transport system |
|  | K02042 | *phnE* | phosphonate transport system, membrane component | phosphonate transport system |
|  | K02044 | *phnD* | phosphonate transport system, periplasmic-binding component | phosphonate transport system |
|  | K05781 | *phnK* | putative phosphonate transport system ATP-binding protein | phosphonate transport system |


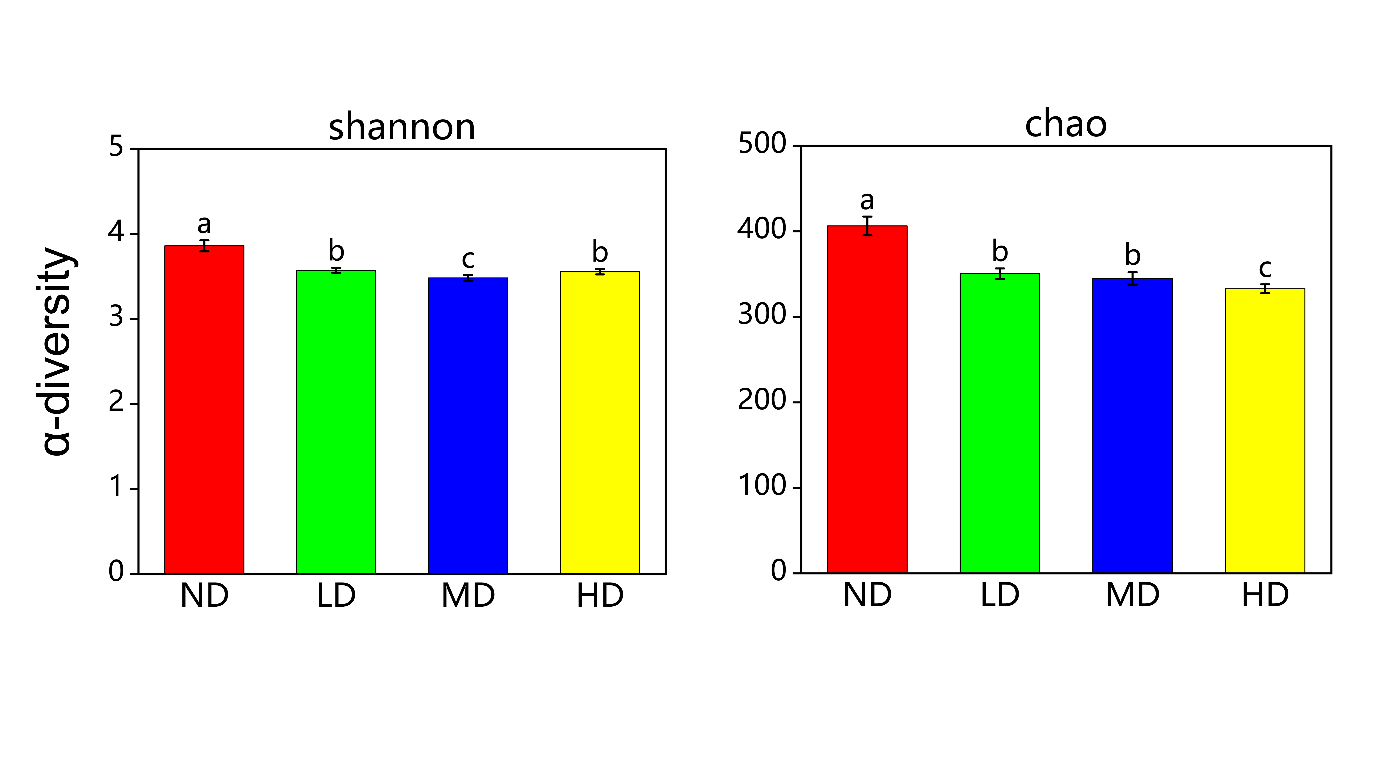
**Figure S1.**

Diversity and richness of P-cycling microbial communities across different degradation levels. Data are reported as mean ± 1 SE (n = 6). Different lowercase letters in the same row mean significant difference at *P* < 0.05. ND, non-degraded wetland; LD, slightly degraded wetland; MD, moderately degraded wetland; HD, heavily degraded wetland.

**Figure S2.
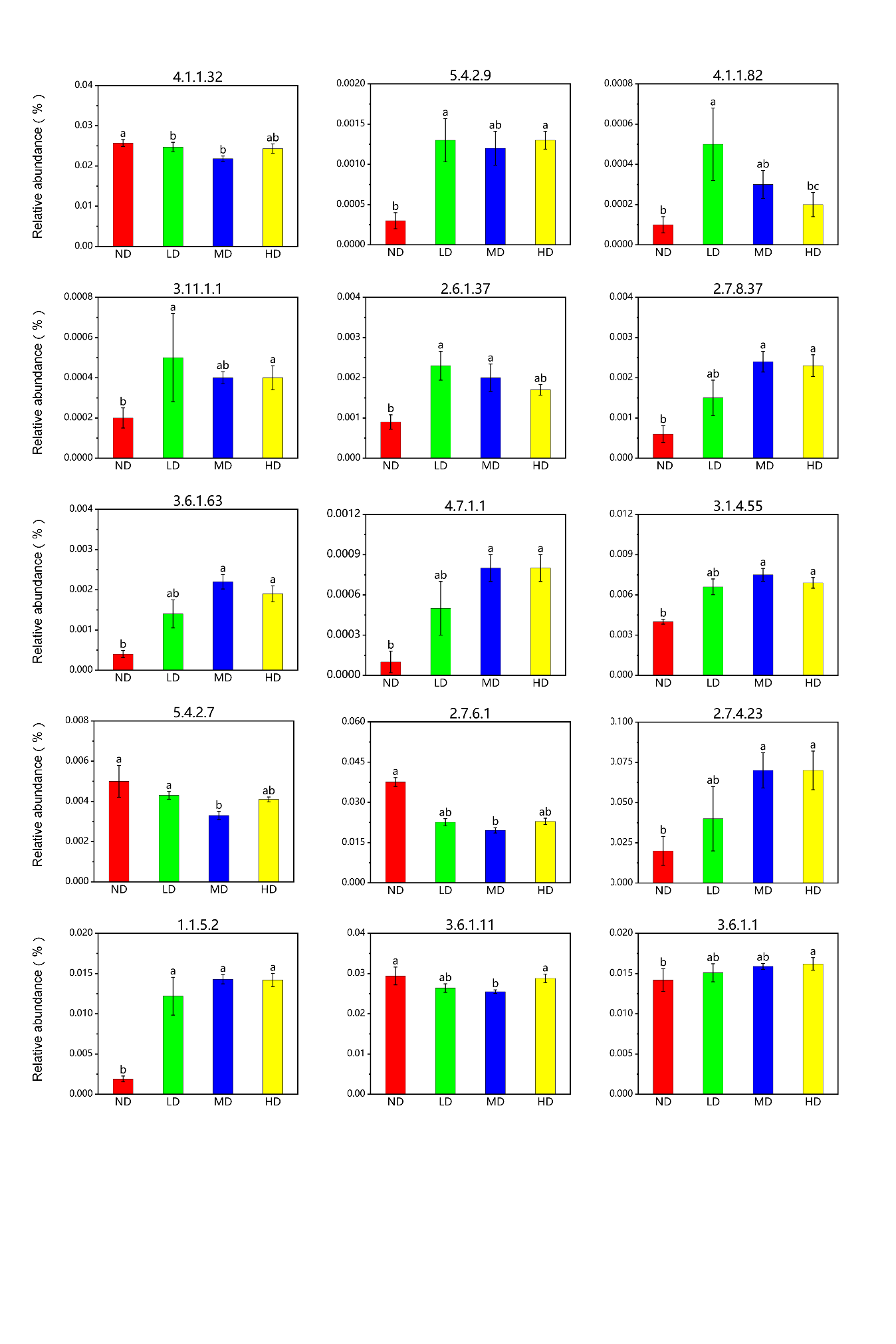
**

Changes in the relative abundance of phosphorus metabolic enzymes across different wetland degradation stages. Data are reported as mean ± 1 SE (n = 6). Different lowercase letters in the same row mean significant difference at *P* < 0.05. ND, non-degraded wetland; LD, slightly degraded wetland; MD, moderately degraded wetland; HD, heavily degraded wetland.

**Figure S3.
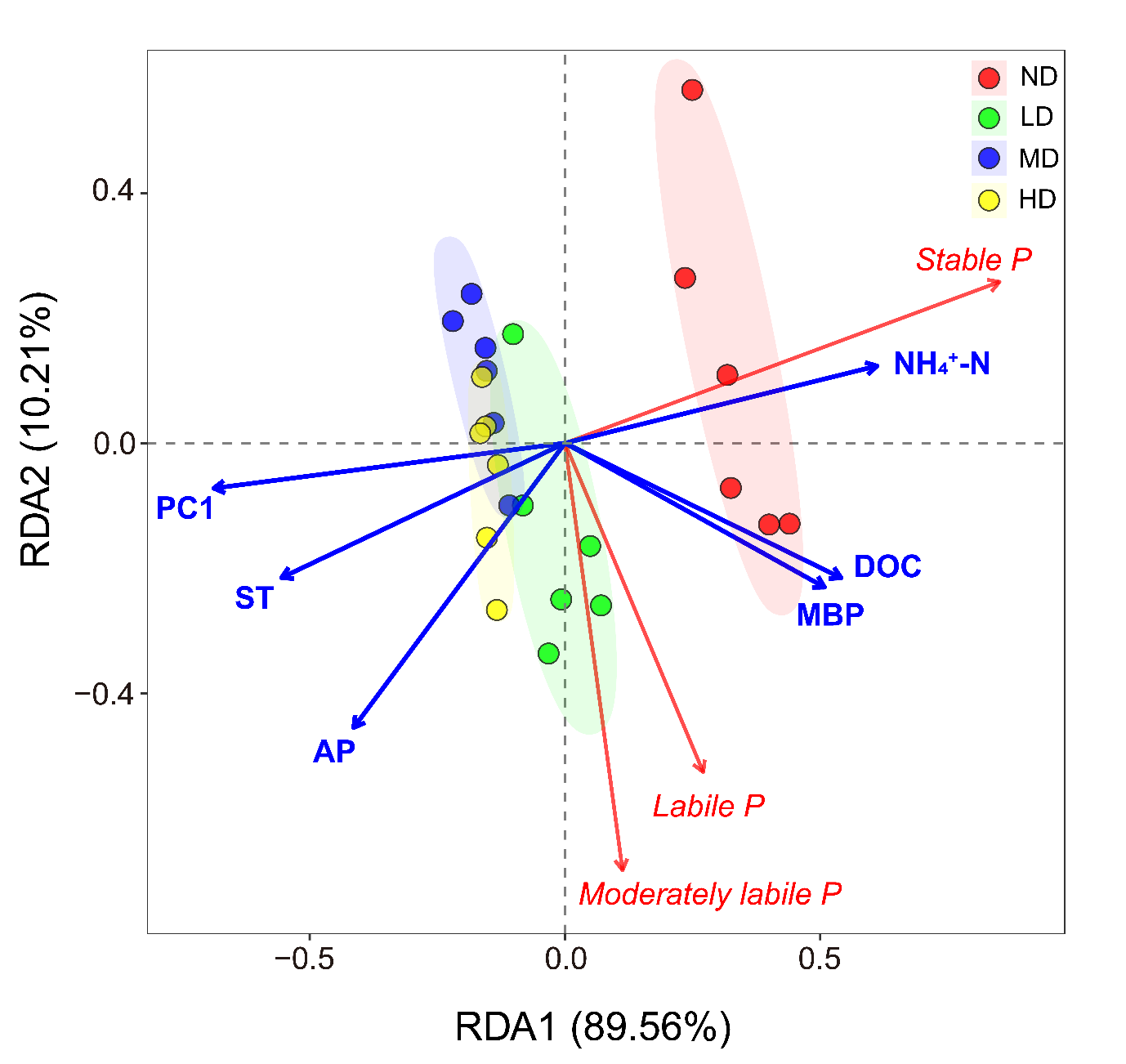
**

Redundancy analysis (RDA) of three major phosphorus fractions (Labile-P, Moderately labile-P, and Stable-P) with environmental factors.

**Figure S4.
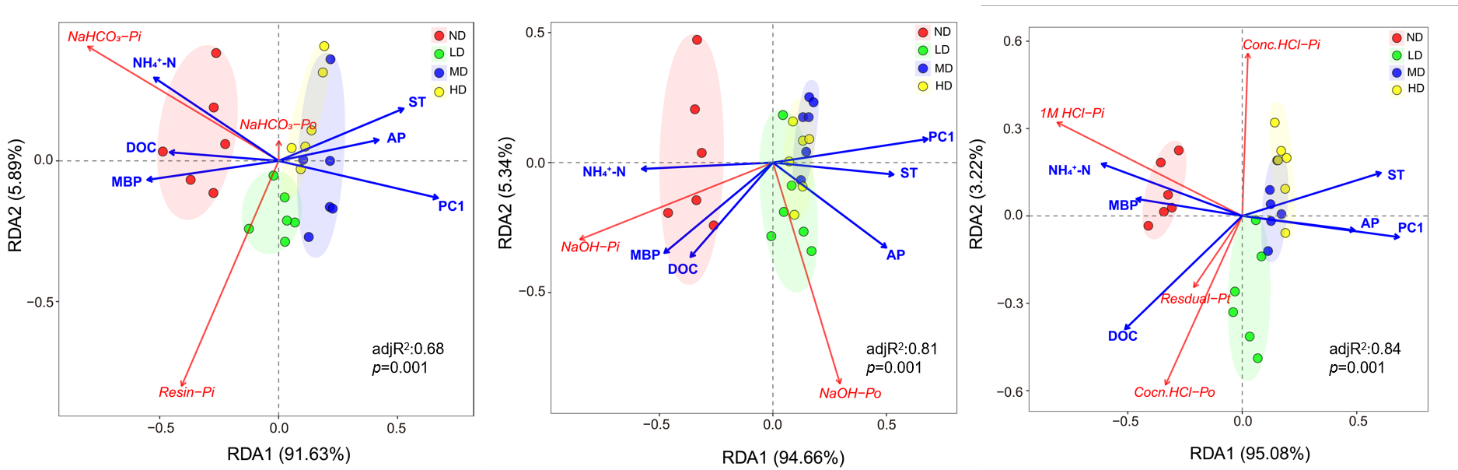
**

Redundancy analysis (RDA) of phosphorus fractions with environmental factors.
